# Adipose tissue mTORC2 regulates ChREBP-driven *de novo* lipogenesis and hepatic glucose metabolism

**DOI:** 10.1038/ncomms11365

**Published:** 2016-04-21

**Authors:** Yuefeng Tang, Martina Wallace, Joan Sanchez-Gurmaches, Wen-Yu Hsiao, Huawei Li, Peter L. Lee, Santiago Vernia, Christian M. Metallo, David A. Guertin

**Affiliations:** 1 Program in Molecular Medicine, University of Massachusetts Medical School, 373 Plantation Street, Worcester, Massachusetts 01605, USA; 2 Department of Engineering, University of California, San Diego, La Jolla, California 92093, USA

## Abstract

Adipose tissue *de novo* lipogenesis (DNL) positively influences insulin sensitivity, is reduced in obesity, and predicts insulin resistance. Therefore, elucidating mechanisms controlling adipose tissue DNL could lead to therapies for type 2 diabetes. Here, we report that mechanistic target of rapamycin complex 2 (mTORC2) functions in white adipose tissue (WAT) to control expression of the lipogenic transcription factor ChREBPβ. Conditionally deleting the essential mTORC2 subunit *Rictor* in mature adipocytes decreases *ChREBPβ* expression, which reduces DNL in WAT, and impairs hepatic insulin sensitivity. Mechanistically, *Rictor*/mTORC2 promotes *ChREBPβ* expression in part by controlling glucose uptake, but without impairing pan-AKT signalling. High-fat diet also rapidly decreases adipose tissue *ChREBPβ* expression and insulin sensitivity in wild-type mice, and does not further exacerbate insulin resistance in adipose tissue *Rictor* knockout mice, implicating adipose tissue DNL as an early target in diet-induced insulin resistance. These data suggest mTORC2 functions in WAT as part of an extra-hepatic nutrient-sensing mechanism to control glucose homeostasis.

## Abstract (web summary)

The kinase mTOR controls anabolic metabolism. Here, the authors create fat-specific mTORC2 knockout mice using the Adiponectin-Cre driver and show mTORC2 signalling is important for systemic metabolic homeostasis by controlling adipocyte *de novo* lipogenesis and glucose uptake.

## Graphical abstract

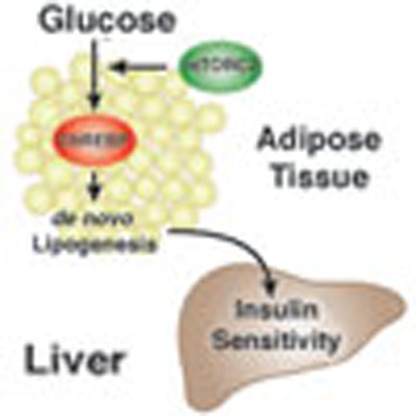

Insulin resistance is a comorbidity of obesity, a risk factor for type 2 diabetes (T2D), and a side effect of the immunosuppressant rapamycin; however, the exact mechanisms that can lead to insulin resistance remain poorly understood. A hallmark of T2D is failure of insulin to suppress hepatic glucose production (HGP) leading to hyperglycemia. In hepatocytes insulin suppresses HGP by stimulating AKT to inhibit FOXO1 (ref. 1). Insulin also regulates HGP in mice, in which hepatic insulin signalling is genetically ablated^2^,^3^,^4^ suggesting the existence of an extrahepatic insulin-sensing tissue that can indirectly control hepatic glucose output. Thus, understanding how organs communicate to control glucose homeostasis is critical to understanding T2D.

Traditionally adipose tissue *de novo* lipogenesis (DNL) was thought to function primarily to store excess energy from carbohydrates as more energy-dense lipid; however, unlike in the liver, in which increased DNL often correlates with insulin resistance, increased DNL in white adipose tissue (WAT) correlates with insulin sensitivity^5^,^6^. Moreover, reduced DNL in WAT is observed in obesity^7^,^8^ and can occur following prolonged rapamycin treatment^9^. Recent work in rodents shows that hepatic insulin resistance develops within a week of high-fat diet (HFD) feeding, coincident with reduced insulin-stimulated glucose uptake into adipose tissue, but without losing insulin-stimulated AKT signalling in fat, and before any detectable decrease in muscle glucose uptake, elevation of lipolysis or inflammation^10^. This supports a model in which HFD causes selective insulin resistance in fat and that adipose tissue glucose uptake and DNL is linked to an extra-hepatic insulin-sensitizing signal that may be targeted early in obesity. Deciphering the upstream mechanisms controlling adipose tissue DNL is therefore critical to understanding the pathogenesis of certain forms of insulin resistance.

DNL involves taking up glucose and converting glucose-derived citrate to acetyl-CoA (by ATP-citrate lyase/ACLY), which is further converted to malonyl-CoA (by acetyl-CoA carboxylase/ACC), and eventually to palmitate (by fatty acid (FA) synthase/FASN). Palmitate is further modified by elongases and desaturases to produce diverse lipids. The transcriptional regulators sterol response element-binding protein 1c (SREBP1c) and carbohydrate response element-binding protein (ChREBP) control lipogenic gene expression^11^,^12^,^13^; however, their distinct regulation and functional roles in adipose tissues are still being worked out. Recently it was shown in adipose tissue that the ChREBPα isoform functions in part by driving expression of the N-terminally truncated ChREBPβ isoform from an alternative promoter^5^. ChREBPβ more potently induces DNL genes and its expression in human adipose tissue correlates with insulin sensitivity^5^,^8^, suggesting ChREBP activity in adipose tissue may be an important regulator of systemic glucose homeostasis.

The mechanistic target of rapamycin (mTOR) kinase is a master regulator of metabolism whose downstream functions are split between at least two distinct complexes. The best understood complex, mTOR complex 1 (mTORC1) is a well-known amino acid and growth factor sensor that promotes anabolic metabolism by driving protein and lipid biosynthesis and suppressing autophagy^14^. Its less understood sibling, mTORC2, is activated by growth factors and is best known as the AKT hydrophobic motif kinase (S473 in AKT1; S474 in AKT2)^15^. It is thought that mTORC2 activity is essential for maximal AKT signalling to its downstream substrates and whether AKT-independent mTORC2 pathways are critical in metabolism is not yet clear. The function of mTORC2 in adipose tissue was previously examined using the aP2-Cre driver to conditionally delete floxed alleles of *Rictor*, an essential subunit of the complex^16^,^17^. However, several recent studies indicate that adiponectin-Cre has greater efficiency and selectivity to mature adipocytes^18^,^19^,^20^,^21^. Thus, the *in vivo* role of mTORC2 in mature adipocytes remains unclear.

We recently reported that mTORC2 regulates expression of lipogenic genes in brown adipose tissue (BAT)^22^ leading us to hypothesize that mTORC2 might also regulate DNL in WAT to control insulin sensitivity. To test this we developed a new fat-specific mTORC2 knockout (KO) model by deleting *Rictor* with adiponectin-Cre. Here, we provide evidence supporting a model in which a primary function of adipocyte mTORC2 is to promote *Chrebpβ* expression and DNL. Moreover, we find that ablating *Rictor*/mTORC2 in WAT alters the lipid composition of fat and causes severe hepatic insulin resistance. Mechanistically, mTORC2 appears to promote *Chrebpβ* expression and DNL at least in part by controlling glucose uptake into adipocytes independently of the classic AKT-AS160 pathway. *Adiponectin-Cre;Rictor* conditional KO mice also exhibit reduced fat growth on a HFD suggesting mTORC2 additionally functions in mature adipocytes to control diet-induced adipose tissue expansion. These data provide a new framework for exploring the role of mTORC2 signalling in obesity and the pathogenesis of insulin resistance.

## Results

### Mice lacking adipocyte *Rictor* have normal body growth

To evaluate the role of mTORC2 specifically in adipose tissue, we generated *Adiponectin-cre;Rictor*^*fl/fl*^ mice (herein *Rictor*^*Adipoq-cre*^ mice). We previously confirmed that adiponectin-Cre targets mature adipocytes with high specificity and efficiency in our colony^23^. Note that significant differences exist between *Rictor*^*Adipoq-cre*^ and mice in which *Rictor* was targeted with *aP2-cre*^16^,^17^. We provide a detailed comparison in the Discussion and in Supplementary Table 1.

**Figure 1:**
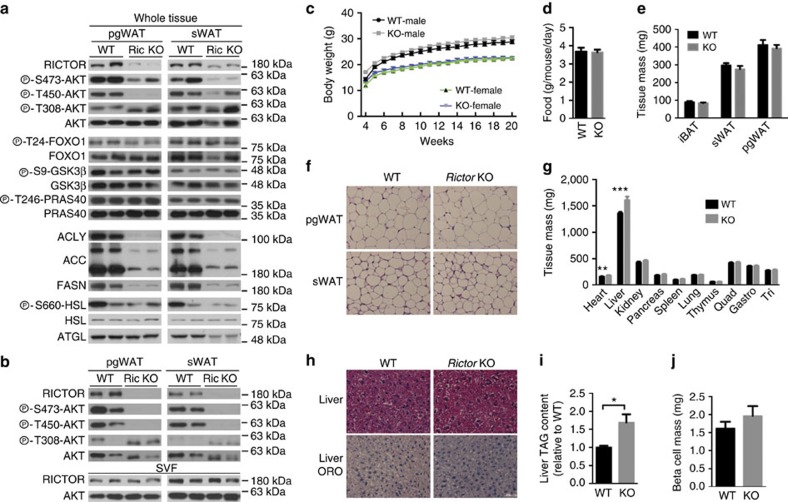
Growth characteristics of mice lacking *Rictor* in adipose tissue. (**a**) Western blots of the indicated total and phosphor-proteins in whole-fat tissue lysates of *Rictor*^*fl/fl*^ (WT) and *Rictor*^*Adipoq-Cre*^ (KO) mice. (**b**) Western blots using lysates of purified adipocytes and the stromal vascular faction (SVF) prepared from pgWAT and sWAT. (**c**) Body growth curves. *n*=8. (**d**) Food consumption. *n*=6. (**e**) Individual fat tissue mass analysis. *n*=8–13. (**f**) Representative H&E images of pgWAT and sWAT. (**g**) Individual lean tissue mass analysis. *n*=8–13. (**h**) Representative H&E images of liver and of liver Oil Red O staining. (**i**) Liver TAG content. *N*=6. (**j**) β-cell mass. *N*=5. Data were analysed by Student's *t*-test. Values are expressed as mean+s.e.m. *P<0.05; **P<0.01; ***P<0.001. Scale bar, 100 μM. H&E, haematoxylin and eosin.

Deleting *Rictor* with adiponectin-Cre greatly reduces RICTOR, pAKT^S473^ and pAKT^T450^ (a growth factor insensitive mTORC2 target site) in the major visceral (that is, perigonadal or pgWAT), subcutaneous (that is, inguinal subcutanteous or sWAT), and brown fat (that is, interscapular BAT) depots (Fig. 1a and Supplementary Fig. 1a) and the residual signal is from stromal vascular fraction (SVF) cells because RICTOR and pAKT^S473/pT450^ is undetectable in purified adipocytes (Fig. 1b). In contrast, pAKT^T308^ is intact (Fig. 1a,b), which maintains AKT's ability to phosphorylate pFOXO1^T24^, pGSK3β^S9^ and pPRAS40^T246^ (Fig. 1a). SVF and hepatic RICTOR levels are normal (Fig. 1b and Supplementary Fig. 1b) confirming targeting specificity.

The body weight (Fig. 1c) and food intake (Fig. 1d) of *Rictor*^*Adipoq-cre*^ mice consuming a standard chow diet does not significantly differ from controls through 20 weeks. The mass of the major fat depots is also unaffected in both male and female mice (Fig. 1e and Supplementary Fig. 1c) and the *Rictor*^*Adipoq-cre*^ adipocytes appear normal by haematoxylin and eosin staining (Fig. 1f and Supplementary Fig. 1d). Liver mass increases in *Rictor*^*Adipoq-cre*^ mice by 18% and 13% in both males and females, respectively (Fig. 1g and Supplementary Fig. 1e). By haematoxylin and eosin stain the liver appears normal (Fig. 1h); however, despite no observable difference in Oil Red O staining in the liver between the control and *Rictor*^*Adipoq-cre*^ KO mice (Fig. 1h) there is a measurable increase in total hepatic TAG content (Fig. 1i) corresponding with a 16% decrease in the number of nuclei per field (Supplementary Fig. 1f) (indicative of increased cell size) that may in part explain the overall increase in liver mass. Heart mass also increases in *Rictor*^*Adipoq-cre*^ KO mice; however, kidney, spleen, lung, thymus and muscle mass is normal (Fig. 1g and Supplementary Fig. 1e). Total pancreas and pancreatic β-cell mass also does not significantly differ between KO and control (Fig. 1j and Supplementary Fig. 1g,h). These observations are in contrast to those reported for *Rictor*^*aP2-cre*^ mice, which have increased total body size because of a global increase in lean tissue mass (including heart, kidney, spleen and pancreas but not the liver) that is attributed to high circulating IGF1 (ref. 16). We find no difference in circulating IGF1 levels between *Rictor*^*Adipoq-cre*^ mice and controls (Supplementary Fig. 1i) and conclude that in 20-week-old mice living under standard conditions, losing adipose tissue *Rictor* does not affect overall fat mass or increase whole-body lean tissue growth in this model.

### Adipocyte mTORC2 controls hepatic glucose production

**Figure 2:**
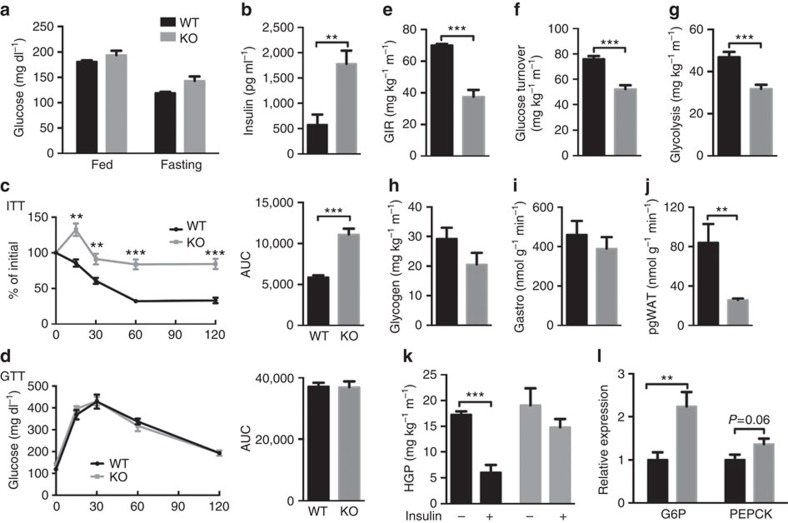
Deleting *Rictor* in fat impairs HGP. (**a**) Plasma glucose level in random fed and 6 h fasted *Rictor*^*fl/fl*^ (WT) and *Rictor*^*Adipoq-Cre*^ (KO) mice. *n*=6 or 8. (**b**) Plasma insulin level in random fed mice. *n*=6. (**c**) Insulin tolerance test results with area under the curve to the right (ITT). *n*=6 or 8 mice. (**d**) Glucose tolerance test results with area under the curve to the right (GTT). *n*=6 or 8 mice. (**e**-**l**) Whole-body glucose homeostasis in 8-10 weeks old male mice was evaluated by hyperinsulinemic-euglycemic clamp (*n*=5 or 6 per group). (**e**) GIR. (**f**) Glucose turnover rate. (**g**) Whole-body glycolysis. (**h**) Whole-body glycogen synthesis. (**i**) Glucose uptake in skeletal muscle (gastro). (**j**) Glucose uptake in adipose tissue (pgWAT). (**k**) HGP without or with insulin stimulation. (**l**) Relative hepatic mRNA expression of the indicated gluconeogenic genes. *N*=8. Data were analysed by Student's *t*-test. Values are expressed as mean+s.e.m. *P<0.05; **P<0.01; ***P<0.001. ITT, insulin tolerance test. GIR, glucose infusion rate. GTT, glucose tolerance test.

Blood glucose concentrations in fasting and fed *Rictor*^*Adipoq-cre*^ mice do not differ from controls; however, this requires approximately threefold higher plasma insulin suggesting insulin resistance (Fig. 2a,b). Insulin intolerance of *Rictor*^*Adipoq-cre*^ mice was supported by insulin tolerance tests (ITTs), showing an 89% increase in AUC (Fig. 2c and Supplementary Fig. 2a); glucose tolerance is normal (Fig. 2d). Acutely inhibiting mTORC2 by treating *Rictor*^*Adipoq-creERT2*^ mice with tamoxifen also ablates RICTOR and pAKT^S473^ within 3-week of treatment (Supplementary Fig. 2b), and this induces insulin intolerance similar to the congenital KOs without affecting glucose tolerance (Supplementary Fig. 2c–e) indicating insulin resistance occurs rapidly with *Rictor* loss.

To better assess insulin action we performed hyperinsulinemic-euglycemic clamps in conscious mice. The steady-state glucose infusion rate required by *Rictor*^*Adipoq-cre*^ mice to maintain euglycemia is 46% lower than controls indicating severe insulin resistance (Fig. 2e). Insulin stimulated whole-body glucose uptake and glycolysis is diminished by 31 and 32%, respectively (Fig. 2f,g), and there is no difference in glycogen plus lipid synthesis (Fig. 2h). Interestingly, insulin stimulated glucose uptake into skeletal muscle—the main site of glucose clearance—is normal (Fig. 2i), while in contrast, insulin stimulated glucose uptake into adipose tissue decreases by 69% in *Rictor*^*Adipoq-cre*^ mice (Fig. 2j). Basal HGP is unaltered between both cohorts; however, insulin fails to suppress HGP in *Rictor*^*Adipoq-cre*^ mice (Fig. 2k). The *Rictor*^*Adipoq-cre*^ mice also express 2.2-fold more hepatic *glucose 6-phosphatase* (*G6P*) (Fig. 2l) and they show less tolerance to a pyruvate bolus (Supplementary Fig. 2f) consistent with increased hepatic gluconeogenesis. Hepatic *phosphoenolpyruvate carboxylase* (*Pepck*) is not significantly elevated (Fig. 2l).

We also examined genes involved in hepatic lipid regulation. Although *Rictor*^*Adipoq-cre*^ livers have elevated TAG content, most lipogenesis genes including hepatic *Srebps* (*Srebf1a*, *Srebf1c* and *Srebf2*), *Chrebpα*, and *Lxrα* express normally in the *Rictor*^*Adipoq-cre*^ KO livers (Supplementary Fig. 2g). We do detect increased *Chrebpβ* expression; however, expression of the ChREBP targets *Acly, Acc* and *Fasn* does not significantly differ (Supplementary Fig. 2h) suggesting ChREBPβ may have a role in gluconeogenesis. We also find increased expression of the TAG synthesis genes *1-acylglycerol-3-phosphate O-acyltransferase 2* (*Agpat2*), whose product converts lysophosphatidic acid to phosphatidic acid in the second step of *de novo* phospholipid synthesis, and *monoacylglycerol O-acyltransferase 1* (*Mgat1*), whose product catalyzes the synthesis of diacylglycerols, in *Rictor*^*Adipoq-cre*^ KO livers (Supplementary Fig. 2i); other TAG synthesis genes are normal. The lipid uptake gene *lipoprotein lipase (Lpl)* also expresses normally in *Rictor*^*Adipoq-cre*^ KO livers; however, *CD36* increases (Supplementary Fig. 2j). Among genes that encode β-oxidation regulators, hepatic *carnitine palmitoyltransferase 1* (*Cpt1)* also increases, but *peroxisome proliferator-activated receptor α* (*Pparα)* and *medium-chain acyl-CoA dehydrogenase* (*Mcad)* express normally (Supplementary Fig. 2k). Collectively, these data suggest that adipose tissue mTORC2 governs production of an adipocyte-derived signal that regulates HGP and lipid handling.

### Intracellular insulin action

**Figure 3:**
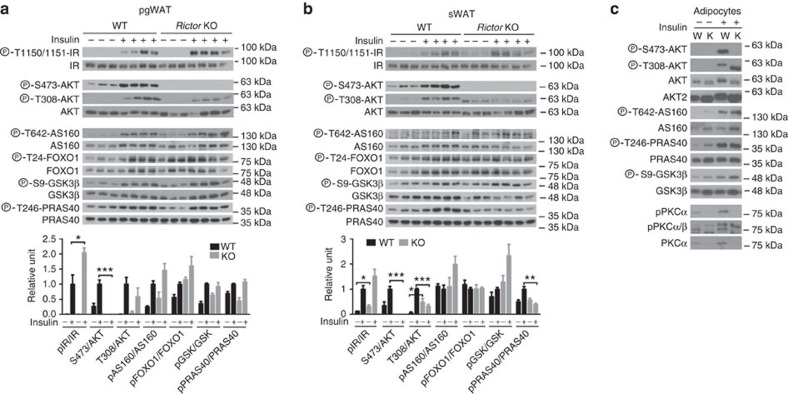
Insulin action in adipose tissues. (**a**,**b**) Western blots of the indicated total and phospho-proteins in whole-tissue lysates prepared from pgWAT (a) and sWAT (b). Male *Rictor*^*fl/fl*^ (WT) and *Rictor*^*Adipoq-Cre*^ (KO) mice (8–10 weeks old) were fasted for 6 h and then injected with insulin (0.75 U kg^−1^) for 15 min before collecting samples. Quantifications are shown below. *n*=3 or 4. Values are expressed as mean+s.e.m. (**c**) Western blot of indicated total and phospho-proteins in mature adipocytes isolated from the pgWAT of *Rictor*^*fl/fl*^ (W) and *Rictor*^*Adipoq-Cre*^ (K) with or without inulin stimulation.

As expected, insulin fails to stimulate AKT^S473^ phosphorylation in *Rictor*^*Adipoq-cre*^ pgWAT while insulin-stimulated pAKT^T308^ is not significantly different (Fig. 3a). Interestingly, activating phosphorylation of the insulin receptor (pIR^Y1150/1151^) is higher in the KO fat (Fig. 3a) possibly suggesting loss of an inhibitory feedback mechanism. Moreover, downstream insulin-stimulated AKT substrate phosphorylation to AS160, FOXO1, GSK3β and PRAS40 does not significantly differ from controls (Fig. 3a) indicating normal insulin-stimulated pan-AKT signalling. Overall, the sWAT gave similar results but with a few noteworthy differences (Fig. 3b). In the mutant sWAT pAKT^T308^ is more resistant to changes in insulin levels (Fig. 3b—top) resulting in increased and decreased phospho-AKT^T308^ signal relative to controls in the fasted and insulin-stimulated state respectively (Fig. 3b—bottom). Phospho-PRAS40 is also reduced only in the mutant sWAT; however, AS160, FOXO1 and GSK3β phosphorylation are normal (Fig. 3c). We confirmed these findings in isolated mature adipocytes (Fig. 3c). The fact that insulin-stimulated IR phosphorylation and AKT signalling is largely intact in the mutant WATs yet insulin fails to efficiently stimulate glucose uptake (Fig. 2j) indicates that *Rictor*^*Adipoq-cre*^ mice have selective adipose tissue insulin resistance.

**Figure 4:**
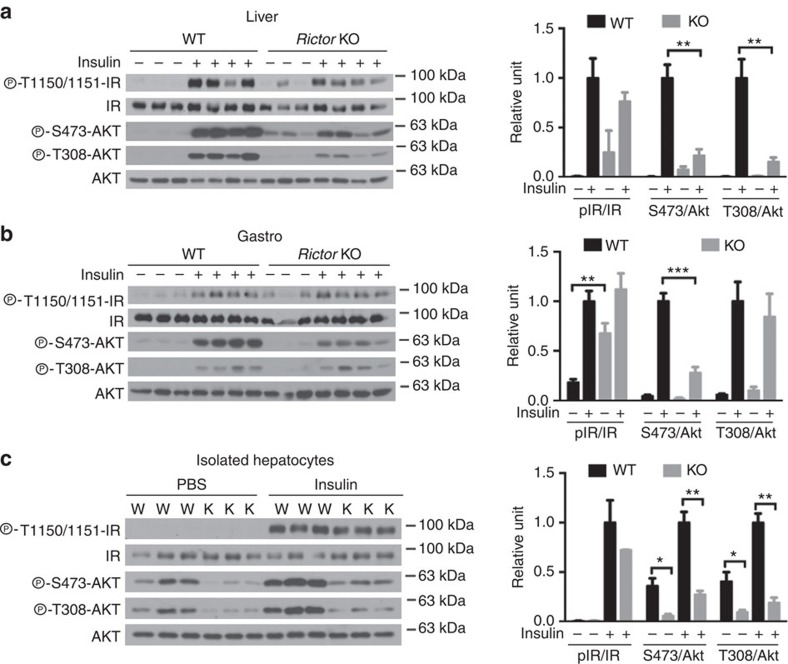
Insulin action in liver and muscle. (**a**,**b**) Western blots of the indicated total and phospho-proteins in whole tissue lysates prepared from liver (a) and skeletal muscle (b). Male *Rictor*^*fl/fl*^ (WT) and *Rictor*^*Adipoq-Cre*^ (KO) mice (8–10 weeks old) were fasted for 6 h and then injected with insulin (0.75 U kg^−1^) for 15 min before collecting samples. (**c**) Western blots of the indicated total and phospho-proteins in isolated hepatocytes. In each panel, the Western blot quantifications are shown to the right. *n*=3 or 4. Values are expressed as mean+s.e.m. *P<0.05; **P<0.01; ***P<0.001.

Insulin-stimulated pAKT^T308^ and pAKT^S473^ are both attenuated in *Rictor*^*Adipoq-cre*^ livers (Fig. 4a) consistent with hepatic insulin-resistance. Curiously, in *Rictor*^*Adipoq-cre*^ muscle IR phosphorylation is slightly higher in the unstimulated state, while insulin-stimulated pAKT^S473^ is blunted (Fig. 4b) suggesting some muscle signalling might also be altered in the mutant mice. However, AKT^T308^ phosphorylation is stimulated normally in *Rictor*^*Adipoq-cre*^ muscle (Fig. 4b), which is consistent with the clamp data showing normal insulin-stimulated muscle glucose clearance (Fig. 2i). These data support a model in which *Rictor* loss in fat most negatively affects hepatic function. Interestingly, primary hepatocytes isolated from *Rictor*^*Adipoq-cre*^ mice maintain insulin resistance even when cultured *ex vivo* (Fig. 4c). Thus, the ‘damage' imposed on hepatic insulin signalling by *Rictor* loss in fat is not easily reversible.

### Altered lipid metabolism and composition in *Rictor*-deficient fat

**Figure 5:**
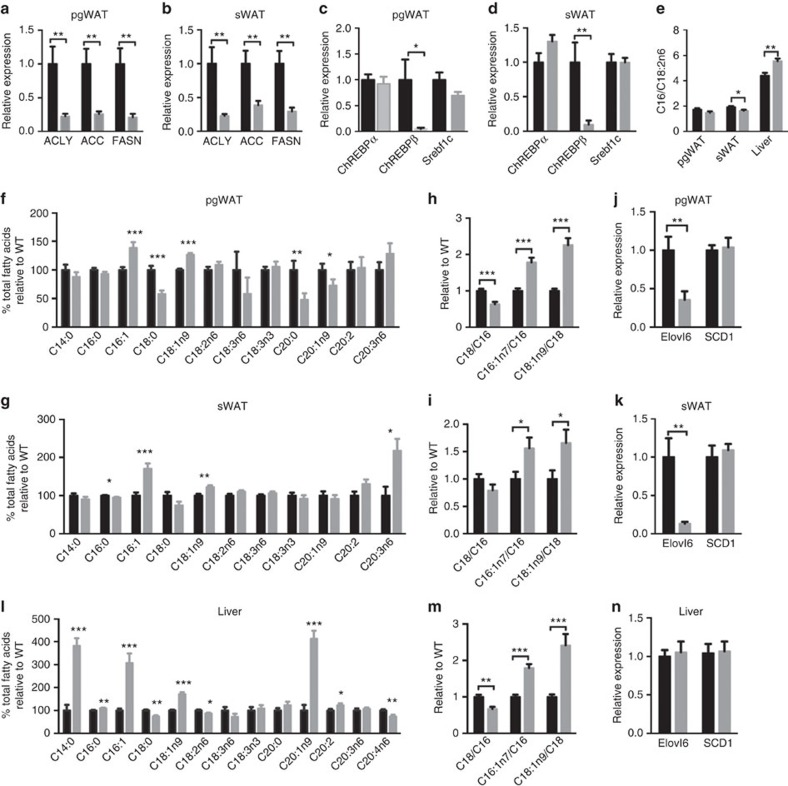
Deleting *Rictor* in adipose tissue reduces DNL and alters the lipid composition of fat and liver. (**a**–**d**) Relative mRNA expression of indicated genes in pgWAT (**a**,**c**) and sWAT (**b**,**d**) *Rictor*^*fl/fl*^ (WT) and *Rictor*^*Adipoq-Cre*^ (KO) mice. *n*=8. (**e**) The ratio of C16/C18:2n6 in pgWAT, sWAT and liver. *n*=8. (**f**,**g**) Lipid profiles of pgWAT (**f**) and sWAT (**g**). *n*=8. (**h**,**i**) The ratio of C18/C16, C18:1n7/C16:1n6 and C18:1n9/C18 in pgWAT and sWAT. *n*=8. (**j**,**k**) Relative mRNA expression of indicated genes in pgWAT (**j**) and sWAT (**k**). (**l**) Lipids profiles of liver. *n*=8. (**m**) The ratio of C18/C16, C18:1n7/C16:1n7 and C18:1n9/C18 in liver. *n*=8. (**n**) Relative mRNA expression of the indicated genes in liver. *n*=8. Data were analysed by Student's *t*-test. Values are expressed as mean+s.e.m. *P<0.05; **P<0.01; ***P<0.001.

**Figure 6:**
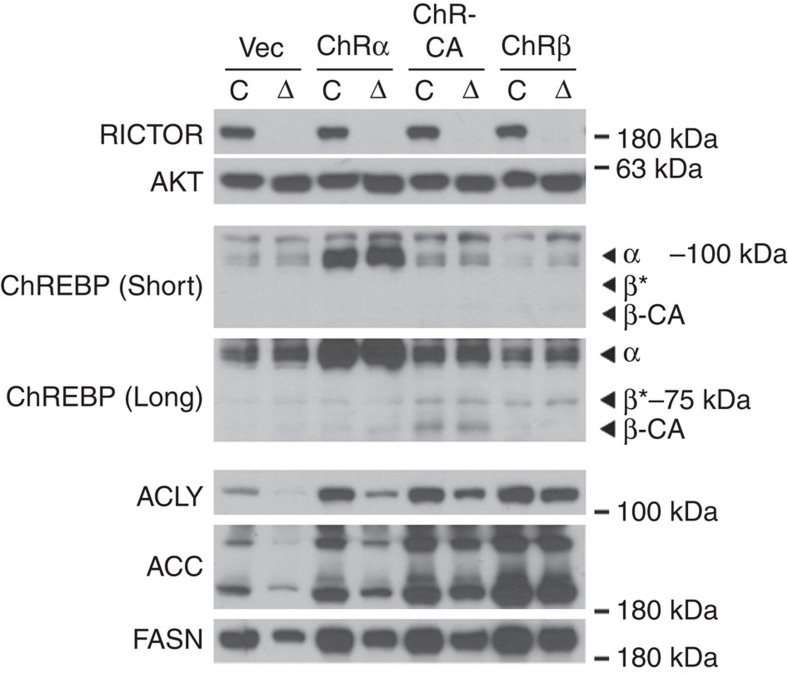
Expressing recombinant ChREBPβ in *Rictor*-deficient adipocytes rescues expression of DNL enzymes. Western blot of indicated proteins in differentiated adipocytes with or without *Rictor* deletion transfected with various rescue constructs that were stably expressed in cells before differentiation. ‘*' indicates ChREBPβ based on molecular weight.

Consistent with the model that DNL in WAT regulates insulin sensitivity^5^,^6^,^24^ we find that adipose tissue *Rictor* loss dramatically reduces *Acly, Acc* and *Fasn* mRNA and protein expression (Figs 1a and 5a,b). The expression of *Chrebpα* and *Srebf1c* expression is unchanged in the mutant fat; however, *Chrebpβ* induction is almost completely blocked in both pgWAT and sWAT (Fig. 5c,d). This is consistent with *Chrebpβ* expression driving DNL in adipocytes^5^. Indeed, overexpressing recombinant ChREBPβ, constitutively active ChREBP^25^ (ChREBP-CA), and to a lesser extent ChREBPα rescues expression of ACLY, ACC and FASN in *Rictor*-deficient adipocytes supporting this notion (Fig. 6). These data implicate adipocyte mTORC2 as a key upstream regulator of ChREBPβ-driven DNL.

Although under standard dietary conditions the adipose tissues predominantly obtain free FAs (FFAs) from the liver and diet rather than DNL, we wondered whether losing *Rictor* in WAT alters FA composition. The ratio of C16:0 (palmitate) to the essential FA C18:2n6 (linoleate)—an index of DNL—is slightly but significantly decreased in *Rictor*^*Adipoq-cre*^ sWAT (Fig. 5e) suggesting that despite the normal size of mutant WATs (though trending smaller) (Fig. 1e) DNL is decreased. Moreover, C18:0 (stearate) levels decrease while C16:1n7 (palmitoleate) and C18:1n9 (oleate) levels increase in *Rictor*^*Adipoq-cre*^ WATs (Fig. 5f,g). These findings are noteworthy because the *de novo* synthesis of palmitoleate by adipose tissue has been linked to improved systemic insulin action^26^. Diets rich in oleate are also reportedly metabolically healthy^27^,^28^. Thus, it does not appear that a palmitoleate or oleate deficiency in fat is causing insulin resistance in *Rictor*^*Adipoq-cre*^ mice.

The altered abundance of very long-chain FAs suggested mTORC2 might additionally regulate FA elongation and/or desaturation (Supplementary Fig. 3a). Elongation of very long-chain FAs 6 (ElOVL6) elongates C16:0 FAs to C18:0 FAs, while steroyl CoA desaturase (SCD1) desaturates C16:0 and C18:0 FAs to C16:1n7 and C18:1n9, respectively. We calculated the ELOVL6 and SCD1 activity ratios by dividing their products by their substrates (Fig. 5h,i), which indicates a decrease and increase respectively in ELOVL6 and SCD1 activity supporting this hypothesis. A broad survey of elongase and desaturase gene expression further indicates that *Elovl6* expression in the WAT of chow-fed mice requires *Rictor* explaining the reduced C18:0/C16:0 ratio (Fig. 5j,k and Supplementary Fig. 3b–d). In contrast, *Scd1* expression is unchanged (Fig. 5j,k). *Elovl6* and *Scd1* are reportedly co-regulated ChREBP targets in the liver^29^. Thus, in adipose tissue *Elovl6* and *Scd1* are not necessarily co-regulated genes and the altered very long-chain FA profile in the WAT of *Rictor*^*Adipoq-cre*^ mice likely reflects defective elongation.

### Deleting *Rictor* in adipose tissue mirrors the effects of HFD

We also examined whether deleting adipose tissue *Rictor* affects hepatic lipid composition. Interestingly, *Rictor*^*Adipoq-cre*^ livers have a lipid profile and ELOVL6/SCD1 ratios that parallel the WATs but without changes in hepatic *Elovl6* or *Scd1* expression (Fig. 5l–n). The livers from *Rictor*^*Adipoq-cre*^ mice also have high levels of C14:0, C16:0, C18:1n9, C20:1n9 and C20:2 FAs with corresponding decreases in C18:2n6 and C20:4n6 FAs (Fig. 5l). The C16:0/C18:2n6 ratio is elevated in *Rictor*^*Adipoq-cre*^ liver (Fig. 5e) consistent with increased DNL. But as indicated above, most of the core hepatic lipogenic genes express normally in the mutant mice (Supplementary Fig. 2g,h) indicating that hepatic lipid remodelling cannot easily be explained by differences in lipid synthesis genes. The hepatic lipid composition of *Rictor*^*Adipoq-cre*^ mice could also be remodeled in part by changes in exogenous lipid uptake as suggested by high hepatic *CD36* expression (Supplementary Fig. 2j). Regardless, the hepatic lipid profile of *Rictor*^*Adipoq-cre*^ mice is remarkably similar to that observed in livers of mice consuming a HFD despite the fact that they are consuming normal chow^30^. This led us to hypothesize that HFD and adipose tissue *Rictor* loss might target a common pathway that antagonizes hepatic metabolism.

**Figure 7:**
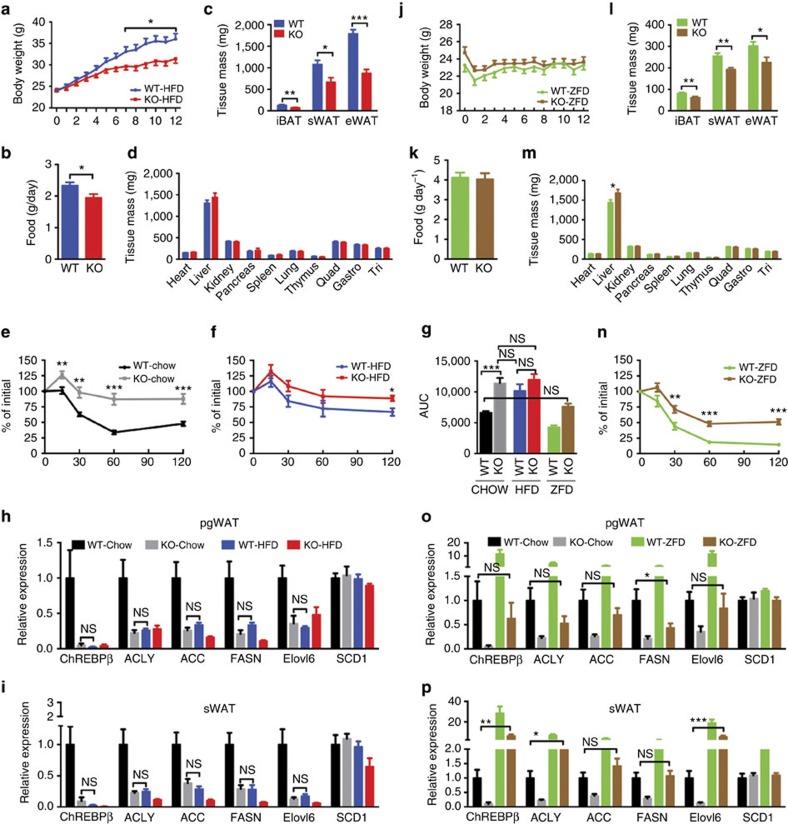
HFD mirrors and ZFD rescues the effects of deleting *Rictor* on DNL and insulin sensitivity. (**a**) Body growth curves of *Rictor*^*fl/fl*^ (WT) and *Rictor*^*Adipoq-Cre*^ (KO) mice under HFD. *n*=8. (**b**) Food consumption under HFD. *n*=5. (**c**) Individual fat tissue mass analysis under HFD. *n*=8. (**d**) Individual lean tissue mass analysis under HFD. *n*=8. (**e**,**f**) Insulin tolerance tests of 20-week-old male mice eating a normal chow diet (**e**) (*n*=9) or a HFD diet (**f**) starting at 8 weeks. *n*=7 mice. (**g**) Area under curve of ITT for **e**,**f** and **n**. Data analysed by two-way ANOVA followed by Tuker's post test. (**h**,**i**) Relative mRNA expression of indicated genes in pgWAT (**h**) and sWAT (**i**) mice consuming chow or HFD. *n*=8 mice. Data were analysed by Student's *t*-test between groups. (**j**) Body growth curves under ZFD. *n*=8. (**k**) Food consumption under ZFD. *n*=5. (**l**) Individual fat tissue mass analysis under ZFD. *n*=8. (**m**) Individual lean tissue mass analysis under ZFD. *n*=8. (**n**) Insulin tolerance tests of 20-week-old male mice eating a ZFD diet starting at 8 weeks. *n*=6 mice. (**o**,**p**) Relative mRNA expression of indicated genes in pgWAT (left) and sWAT (right) of mice consuming chow or ZFD. *n*=8 mice. Data were analysed by Student's *t*-test between groups. Values are expressed as mean+s.e.m. *P<0.05; **P<0.01; ***P<0.001. ITT, insulin tolerance test.

To explore this further, we compared the effects of HFD to deleting *Rictor* in adipose tissue by feeding both WT and *Rictor*^*Adipoq-cre*^ mice a HFD for 12 weeks. Interestingly, *Rictor*^*Adipoq-cre*^ mice consuming HFD are resistant to weight gain (Fig. 7a). This is due to a defect in adipose tissue expansion that may partly result from a slight reduction in food consumption (Fig. 7b,c). This is in stark contrast to *Rictor*^*aP2-Cre*^ mice, which gain more total body weight, more pgWAT mass and more lean tissue mass on HFD^16^.

Importantly, there is no significant difference in insulin tolerance between *Rictor*^*Adipoq-cre*^ mice consuming chow and age-matched WT mice consuming HFD (Fig. 7e–g). Feeding WT mice a HFD also decreases *Chrebpβ*, *Acly*, *Acc*, *Fasn* and *Elovl6* expression in both the pgWAT and sWAT to the same level as that which is observed in the chow-fed *Rictor*^*Adipoq-cre*^ mice (Fig. 7h,i). This is also reflected in the ACLY, ACC and FASN protein expression patterns particularly in the sWAT (Supplementary Fig. 4a,b). In addition, the HFD does not further exacerbate insulin resistance in *Rictor*^*Adipoq-cre*^ mice despite these mice developing additional hepatic steatosis (Fig. 7f,g and Supplementary Fig. 4c). Thus, HFD and adipose tissue *Rictor* loss have similar affects on gene expression and insulin sensitivity suggesting they may target a common pathway.

**Figure 8:**
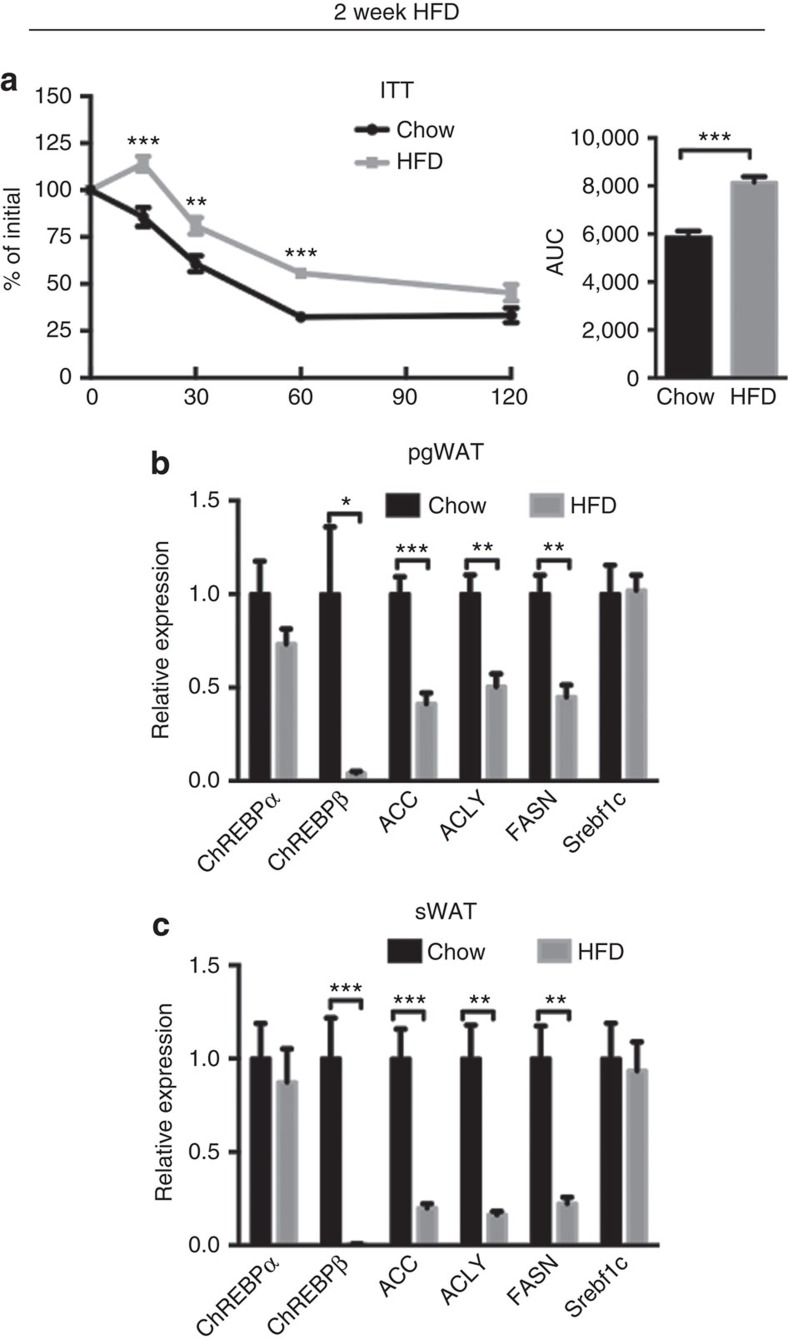
HFD induce insulin resistance and repress DNL for a short treatment. (**a**) ITT of wild-type mice under chow or HFD for 2 weeks started at 8 weeks old. (**b**,**c**) The relative mRNA expression of indicated genes in pgWAT (**b**) and sWAT (**c**) of mice under HFD for 2 weeks. *n*=5. Data were analysed by Student's *t*-test. Values are expressed as mean+s.e.m. *P<0.05; **P<0.01; ***P<0.001. ITT, insulin tolerance test.

To test whether the adipocyte mTORC2-DNL pathway might be an early target of obesity-induced insulin resistance, we placed WT mice on HFD for only 2 weeks, which induces mild insulin resistance (Fig. 8a) and examined lipogenic gene expression. After only 2 weeks HFD, *Chrebpβ, Acly, Acc* and *Fasn*, but not *Srebf1c* gene expression decreases in pgWAT and even more dramatically in the sWAT of WT mice (Fig. 8b,c). These data are consistent with adipose tissue mTORC2 regulating DNL and insulin sensitivity by a mechanism that may be an early target of obesity.

### A lipogenic diet improves the insulin sensitivity of knockout mice

We next placed *Rictor*^*Adipoq-cre*^ mice on a high carbohydrate/zero-fat diet (ZFD) for 12 weeks to maximize effects caused by a DNL deficiency. *Rictor*^*Adipoq-cre*^ mice consuming a ZFD maintain a body weight similar to controls and consume the same amount of food (Fig. 7j,k). However, the adipose tissues are smaller (Fig. 7l) indicating that *Rictor* is also more critical for fat growth with increasing carbohydrate load. Liver mass also increases in ZFD-fed mutants (Fig. 7m) explaining why total body mass is unchanged relative to controls despite the decrease in fat mass.

Interestingly, consuming a ZFD restores insulin tolerance in *Rictor*^*Adipoq-cre*^ mice back to the level observed in the benchmark chow-fed controls (Fig. 7g,n). This correlates with restoration of most lipogenic genes (for example, *Chrebpβ*, *Acly*, *Acc* and *Elovl6*) back to the benchmark expression level (that is, not significantly different from chow-fed controls) (Fig. 7o). ZFD similarly restores sWAT lipogenic gene expression, and for *Chrebpβ*, *Acly* and *Elovl6*, to levels even higher than in the sWAT of chow-fed controls (Fig. 7p). These increases are mirrored by increases in ACLY, ACC and FASN protein expression (Supplementary Fig. 5a,b). Notably, while ZFD increases lipogenic gene expression in *Rictor*^*Adipoq-cre*^ mice to chow-fed levels, lipogenic gene expression still remains lower relative to the ZFD-fed control group (Fig. 7o,p). In fact, lipogenic gene expression responds robustly in the ZFD-fed control group exemplified by ∼11- and 28-fold *Chrebpβ* induction in pgWAT and sWAT, respectively, over chow-fed controls (Fig. 7o,p). Thus, even though a ZFD improves insulin sensitivity in *Rictor*^*Adipoq-cre*^ mice (presumably by ‘forcing' more glucose into adipocytes), the lipogenic genes do not respond at full capacity. Notably, ZFD also increases hepatic lipogenic gene expression and steatosis in both the control and *Rictor*^*Adipoq-cre*^ mice (Supplementary Fig. 5c,d). This is consistent with high ChREBP expression in the liver increases hepatic lipogenic gene expression and steatosis without impairing insulin sensitivity^29^. Collectively, these data support a model in which adipose tissue mTORC2 regulates lipogenic gene expression to produce an insulin-sensitizing signal.

### Reduced glucose uptake and DNL in *Rictor*-deficient adipocytes

**Figure 9:**
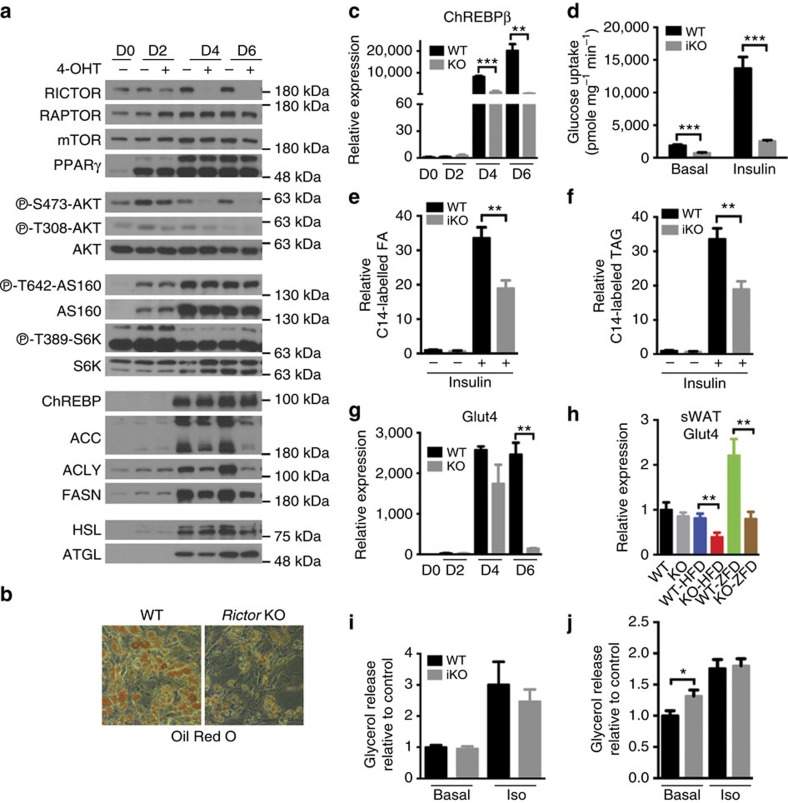
Decreased glucose uptake and Chrebpβ-driven DNL is a primary consequence of *Rictor* loss in adipocytes. (**a**) Western blots of indicated proteins at different days of differentiation using *Rictor iKO* primary adipocytes (described in Methods). (**b**) Oil Red O staining of differentiated adipocytes. (**c**) The relative mRNA level of *ChREBPβ* in differentiated cells at various time points. *n*=3. (**d**) 2-DG uptake in differentiated adipocytes without or with insulin stimulation. *n*=3. (**e**) The C14-glucose-derived FA in differentiated adipocytes. *n*=3. (**f**) The C14-glucose derived triglyceride (TAG) in differentiated adipocytes. *n*=3. (**g**) Relative *glut4* expression in differentiated cells at various time points. *n*=3. (**h**) Relative *glut4* expression in sWAT. *n*=8. (**i**) Glycerol release in differentiated adipocytes under basal and isoproterenol (Iso) stimulation. *n*=4. (**j**) Glycerol release in *ex vivo* pgWAT under basal and isoproterenol stimulation. *n*=6. Data were analysed by Student's *t*-test. Values are expressed as mean+s.e.m. *P<0.05; **P<0.01; ***P<0.001.

To better define the mechanism by which adipose tissue *Rictor* loss alters adipocyte function, we generated primary white preadipocytes harboring a *Rictor* (*UBC-CRE*^*ERT2*^) inducible**-KO (*Rictor-iKO*) system to examine the acute effects of inhibiting mTORC2 on differentiation and function. When compared with their isogenic controls, primary *Rictor-iKO* preadipocytes differentiate normally based on PPARγ, ChREBPα, *C/ebpα*, *C/ebpβ*, *Ap2* and *Adiponectin* expression (Fig. 9a and Supplementary Fig. 6a). In high-glucose culture medium, DNL is the primary driver of lipid droplet formation and under these conditions *Rictor-iKO* cells have smaller lipid droplets (Fig. 9b). *Rictor-iKO* cells also fail to upregulate *Chrebpβ* mRNA and *Acly, Acc* and *Fasn* mRNA and protein (Fig. 9a,c), consistent with a DNL deficiency. Moreover, both basal and insulin-stimulated glucose uptake as well as glucose incorporation into FFAs and TAGs are blunted in *Rictor-iKO* cells (Fig. 9d,e,f). As expected, RICTOR is completely ablated in the *Rictor-iKO* cells by day 6 of differentiation at which point AKT^S473^ phosphorylation is also undetectable (Fig. 9a). In contrast, phosphorylation of AKT^T308^, AS160^T642^ and S6K^T389^ (a measure of mTORC1 activity) is unchanged relative to the isogenic control (Fig. 9a). Thus, decreased glucose uptake and DNL is primary consequence of *Rictor* loss.

GLUT4 is the major glucose transporter for insulin-stimulated glucose uptake into adipocytes. Therefore, we next examined whether GLUT4 regulation is defective in *Rictor*-deficient adipocytes. Indeed, in primary *Rictor-iKO* cells, *Glut4* mRNA and protein fail to induce normally during differentiation (Fig. 9g and Supplementary Fig. 6b). Similarly, in mice consuming the lipogenic ZFD, *Glut4* is also reduced in both the sWAT and pgWAT of *Rictor*^*Adipoq-cre*^ mice (Fig. 9h and Supplementary Fig. 6c). Thus, in highly lipogenic conditions such as in culture medium and a high-carbohydrate diet, *Rictor* is required for maximal *Glut4* gene expression. In mice consuming normal chow however, *Glut4* expression does not significantly differ in either depot from controls (Fig. 9h and Supplementary Fig. 6c,d). Moreover, in HFD-fed mice *Glut4* expression only decreases in the mutant sWAT and not pgWAT (Fig. 9h and Supplementary Fig. 6c). Thus, additional mechanism(s) of glucose regulation by *Rictor*/mTORC2 in adipocytes likely exist.

We also considered other proposed modulators of insulin resistance, such as increased lipolysis. Primary *Rictor-iKO* adipocytes show no difference in basal or isoproterenol-stimulated glycerol release (Fig. 9i) arguing against increased lipolysis as a primary target of *Rictor* loss. However, pgWAT depots resected from *Rictor*^*Adipoq-cre*^ mice show a modest increase in basal glycerol release *ex vivo*; isoproterenol-stimulated glycerol release is normal (Fig. 9j). When administered at a controlled concentration, insulin is also ineffective at suppressing circulating FFA levels *in vivo* in fasted *Rictor*^*Adipoq-cre*^ mice (Supplementary Fig. 6e), which is consistent with insulin resistance but could reflect liver dysfunction. High cholesterol is detected in the mutants, however, circulating FFA and TAG levels are not significantly different between fasted control and *Rictor*^*Adipoq-cre*^ mice possibly due to the high basal insulin levels (Supplementary Fig. 6f–h). Moreover, *Rictor*^*Adipoq-cre*^ mice have normal hormone sensitive lipase (HSL) levels in both depots and increased HSL phosphorylation is not detected (Fig. 1a). Higher levels of adipose triglyceride lipase (ATGL) associate with increased lipolysis; however, ATGL levels are also normal in pgWAT and lower in sWAT (Fig. 1a). Thus, defective lipolysis may contribute to insulin resistance in the prolonged absence of *Rictor*/mTORC2 in fat but it does not appear to be a primary effect.

**Figure 10:**
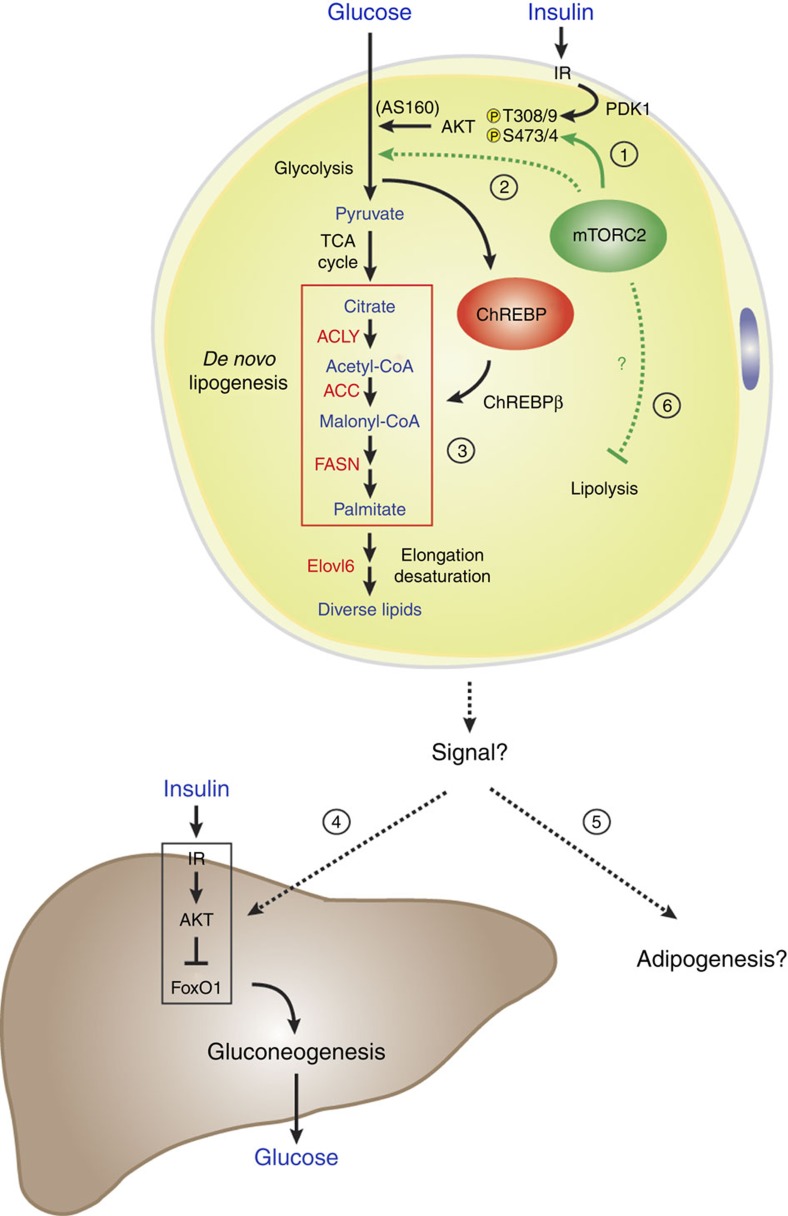
A model of RICTOR/mTORC2 function in adipocytes. (1) In adipocytes, mTORC2 phosphorylates AKT in the hydrophobic motif (S473 in AKT1; S474 in AKT2) while PDK1 phosphorylates AKT in the kinase domain (T308 in AKT1; T309 in AKT2), which promotes maximal AKT activity. This includes stimulating GLUT4 translocation to the plasma membrane by inhibiting AS160. (2) By deleting *Rictor* in mature adipocytes, we provide evidence that *Rictor*/mTORC2 is not essential for AKT signalling to AS160 and other classic substrates, but it is required for normal glucose uptake and ChREBP activity. This suggests mTORC2 might only be essential for a specific AKT-target other than AS160 that promotes glucose uptake; or alternatively, mTORC2 may regulate glucose uptake by an AKT-independent mechanism (also see Discussion). (3) Furthermore, mTORC2-dependent glucose uptake drives ChREBP-dependent DNL and the production of a signal(s) that (4) promotes hepatic insulin sensitivity and possibly (5) HFD-induced adipogenesis. (6) Prolonged *Rictor* loss may lead to additional defects such as increased lipolysis.

TNF-α is undetectable in *Rictor*^*Adipoq-cre*^ mice and leptin, resistin and PAI-1 levels are normal (Supplementary Fig. 6i–k). We do detect reduced plasma adiponectin by ∼32% (Supplementary Fig. 6l), which could contribute to insulin resistance. However, reducing adiponectin levels reportedly has no or only a mild defect in insulin sensitivity in chow-fed mice^31^,^32^ suggesting this alone likely does not explain the severe insulin resistance of *Rictor*^*Adipoq-cre*^ mice. Thus, based on the collective *in vivo* and *in vitro* primary cell data, we propose that a primary function of mTORC2 in adipocytes is to control ChREBP activity by regulating glucose flux, which promotes DNL and the production of a signal(s) that promote insulin sensitivity and possibly adipogenesis (Fig. 10). However, prolonged *Rictor*/mTORC2 loss may lead to secondary metabolic changes that exacerbate the phenotype.

## Discussion

We describe a novel model of mTORC2 in adipose tissue based on conditional deletion of *Rictor* with adiponectin-Cre. There are several phenotypic differences compared with mice in which *Rictor* was targeted with aP2-Cre^16^,^17^. A detailed comparison can be found in Supplementary Table 1. These differences likely reflect the higher efficiency and specificity of adiponectin-Cre^18^,^19^,^21^,^33^. For example, aP2-Cre incompletely targets adipocytes and additionally targets adipose tissue endothelial cells^33^. We find that *Rictor*^*Adipoq-Cre*^ mice exhibit more severe insulin resistance. In addition, *Rictor*^*Adipoq-Cre*^ gain less weight than controls on HFD while in contrast *Rictor*^*aP2-Cre*^ mice gain more weight on HFD. The reduced weight of *Rictor*^*Adipoq-Cre*^ mice on HFD is largely due to a decrease in fat mass, but whether resistance to obesity reflects the deficiency in *Chrebpβ* expression or DNL is not yet clear. Interestingly, it was suggested recently that ChREBP may promote PPARγ activity by controlling the synthesis of FAs that function as PPARγ ligands^25^. In addition, mice lacking *Fasn* in adipose tissue are also resistant to HFD and this is attributed to defective synthesis of a PPARγ ligand^34^. Such a ligand could act in a paracrine manner to stimulate new adipogenesis. This requires further investigation.

Based on our results we hypothesize that adipose tissue mTORC2 functions as part of an extra-hepatic nutrient-sensing mechanism that relays the organism's nutritional state to the liver to control insulin sensitivity and glucose homeostasis (Fig. 10). Mechanistically, our data suggest that adipocyte mTORC2 controls ChREBP activity and DNL at least in part by regulating glucose flux. Interestingly however, this mTORC2 function appears to operate independently of AKT, the canonical mTORC2 substrate. One possibility is that following *Rictor* deletion, AKT signalling reprograms to overcome mTORC2-dependency for some functions; however, for other AKT substrates the dependency for mTORC2 cannot be overcome. An alternative possibility is that only some AKT substrates require mTORC2-dependent hydrophobic motif phosphorylation (S473 in AKT1; S474 in AKT2). A third possibility is that AKT-independent mTORC2 pathways also control glucose flux, although these possibilities are not mutually exclusive.

A classic mechanism by which insulin stimulates glucose uptake is by promoting AKT-dependent phosphorylation of AS160, which facilitates GLUT4 translocation to the plasma membrane^35^. However, AS160 phosphorylation is normal in *Rictor*-deficient adipocytes. Whether adipose tissue mTORC2 regulates GLUT4 translocation *in vivo* by other mechanism(s) needs further investigation^36^. One pathway suggested by our data is that mTORC2 might control glucose uptake by controlling *Glut4* transcription. This function of mTORC2 appears to be most essential when the glucose load is high (for example, culture medium or when mice are consuming ZFD/high-carbohydrate diet). However, another mechanism(s) must exist because *Glut4* expression is unchanged in the fat of *Rictor*^*Adipoq1-Cre*^ mice consuming normal chow. One possibility based on work in glioblastoma cells is that mTORC2 may regulate expression of glycolytic enzymes independently of AKT; however, the mechanism is not clear^37^. Nevertheless, our findings suggest that selective mTORC2 activators may be useful anti-diabetic drugs.

Although it is well known that in the pathogenesis of T2D selective insulin resistance occurs in the liver^38^ (in which insulin fails to suppress gluconeogenesis but continues to promote lipogenesis) it is becoming increasingly clear that selective insulin resistance also occurs in adipose tissue^10^,^39^,^40^,^41^. However, this has been difficult to understand due to lack of a genetic model. Adipocytes lacking *Rictor* demonstrate selective insulin resistance in that only insulin-stimulated glucose uptake and not insulin-stimulated AKT signalling is impaired. An alternative interpretation is that *Rictor*-deficient adipocytes are not insulin resistant *per se*, but rather have impaired glucose uptake through an insulin/AKT-independent mechanism that indirectly attenuates insulin-stimulated glucose flux. Regardless, the *Rictor*^*Adipoq-cre*^ mice provide a novel model of selective insulin resistance in adipose tissue that will be useful for understanding human selective insulin resistance.

What is the adipocyte-derived signal that communicates with the liver? One possibility based on recent evidence is that DNL in adipose tissue might generate a specific bioactive lipid(s) or other factor that functions as an insulin-sensitizer^6^,^26^. Alternatively, the signal from the fat might travel indirectly to the liver via another tissue. For example, high basal insulin levels could promote insulin resistance. Nevertheless, our results support an emerging model in which adipocyte-derived signals, possibly specific *de novo* synthesized lipids, are critical in regulating systemic insulin sensitivity^2^,^3^,^5^,^6^,^26^,^42^,^43^,^44^. Notably, we observe that lipogenic gene expression is rapidly downregulated following a switch to HFD suggesting DNL may be an early target in pathogenesis of diet-induced insulin resistance.

Is there a role for lipolysis in *Rictor*^*Adipoq-cre*^ mice? We did not detect a lipolysis defect in primary cells suggesting altered lipolysis may not be a primary effect of *Rictor* loss. However, tissue explants from the *Rictor*^*Adipoq-cre*^ mice exhibit an increase in glycerol release suggesting elevated lipolysis likely contributes eventually to the *in vivo Rictor*^*Adipoq-cre*^ phenotype. Elevated lipolysis would increase FA flux to the liver, which was recently shown to be a mechanism of impairing HGP^45^. Notably, a recent reevaluation of the literature reveals that insulin resistance exists in human obesity without elevated FFAs, and that elevated FFAs do not necessarily cause insulin resistance^46^. Moreover, mice consuming a HFD for only a few days have impaired glucose uptake into fat and impaired HGP without altered plasma FFA levels or reduced muscle glucose uptake, and without inflammation or altered adipokine secretion^10^. Nevertheless, the use of inducible KO models and *in vivo* metabolomics is required to define progressively the pathogenesis of hepatic insulin resistance in *Rictor*^*Adipoq-cre*^ mice.

A complication after organ transplantation is a syndrome called new onset diabetes after transplantation^47^,^48^. Immunosuppressants such as rapamcyin associate with new onset diabetes after transplantation, and in rodent models, rapamycin causes glucose intolerance and insulin resistance^49^,^50^,^51^; however, the mechanism of rapamycin-induced metabolic disease is unresolved. Rapamycin's ability to inhibit hepatic mTORC2 may explain why it causes glucose intolerance because mice lacking hepatic *Rictor* but not *Raptor* are glucose intolerant^52^; however, hepatic *Rictor*-deficient mice have relatively normal insulin sensitivity indicating unknown extra-hepatic target(s) likely contribute to rapamycin-induced metabolic syndrome. Our genetic model suggests that targeting adipose tissue mTORC2 may be one mechanism by which rapamycin causes insulin resistance. However, rapamycin's effect on fat metabolism is likely complex and fully understanding it will require carefully analysing the acute and chronic effects of inhibiting each mTOR complex versus rapamycin in different fat depots.

In this study, we report a novel mouse model of mTORC2 loss selectively in adipocytes. Our results provide a new framework for studying nutrient and growth factor sensing pathways in adipose tissue metabolism and their role in organ-to-organ communication networks, which may have important implications for understanding and treating human pathologies associated with obesity and lipodystrophy.

## Methods

### Mice

*Rictor*-floxed mice were described previously^53^ and backcrossed with C57BL/6 for 10 generations. Floxed mice were crossed with mice expressing either adiponectin-Cre or adiponetin- Cre^ERT2^, or with Ubc-Cre^ERT2^ (JAX #007001) mice to generate conditional or inducible KO models. Floxed Cre-negative mice were used as wild-type controls. Mice were kept on a daily 12 h light/dark cycle and fed a normal chow diet (Prolab Isopro RMH 3000) from Lab Diet *ad libitum* at 22 °C. All animal experiments were approved by the University of Massachusetts Medical school animal care and use committee. For the inducible CreER models, *Rictor*^*fl/fl*^ (WT) and *Adiponetin-CreERT2;Rictor*^*fl/fl*^ (iKO) male mice at 8 weeks old were treated with 3 mg Tamoxifen per day (i.p.) for 6 constitutive days. The age of the mice used for all studies were 8–20 weeks old. For 2 weeks HFD experiment, mice were randomly placed into cages with chow or HFD. No other randomization was used while conducting experiments. No animals were excluded from any experiments, unless they displayed obvious wounds from fighting as determined by our veterinarians

All animal studies were designed to minimize and control for confounding variables such as mouse gender and age. Based on our previous studies, we use a minimum of six animals per treatment group to achieve statistical power to detect significant differences when measuring RNA, tissue mass, body weight and blood metabolites. Researchers were not blinded to the genotype.

### Antibodies and reagents

AS160 (07-741) was purchased from Millipore. PPARγ (sc-7196) and p70 S6K (sc-9027) are from Santa Cruz. ChREBP (NB400-135) is from Novus Biologicals. All other antibodies including Rictor (2140), Raptor (2280), HSL (4107), ATGL (2439), PRAS40 (2691), AKT (9272), GSK3β (9315), ACC (3676), ACLY (4332), FASN (3180), IR (3025), S473-AKT (4058), T308-AKT (4056), T24-FoxO1 (9464), T389-S6K (234), p-IR (3024), T246-pPRAS40 (2997), S660-pHSL (4126) and T642-pAS160 (4288), were purchased from Cell Signaling Technologies. 4-hydroxy-tamoxifen (4-OHT) was obtained from Toronto Research Chemicals. Rapamycin was purchased from LC Laboratories. Dexamethasone, 3-isobutyl-1-methylxanthine (IBMX), Tamoxifen, and all other reagents were from Sigma-Aldrich.

### Metabolic Studies

Hyperinsulinemic-euglycemic clamps were performed following an overnight fast, a 2-h hyperinsulinemic (insulin at 150 mU kg^−1^ body weight priming followed by 2.5 mU kg^−1^ min^−1^)-euglycemic clamp was conducted in awake mice using (3-3H)-glucose and 2-deoxy-D-(1-14C)-glucose to assess glucose metabolism in individual tissues as described previously^54^. At 8 weeks of age, male mice were fed a normal chow diet (Prolab Isopro RMH 3000) from Lab Diet, 60% HFD (D12492 Harlan Laboratories) or high-carbohydrate ZFD (TD.03314 Harlan Laboratories) and monitored for 12 weeks. Body weight was recorded weekly. The analysis of blood metabolites was performed by at the Joslin Diabetes Center (Boston). For glucose tolerance tests and pyruvate tolerance tests, mice were fasted overnight (16 h) and then administrated 2 g kg^−1^ of body weight of glucose or sodium pyruvate by intraperitoneal (i.p.) injection. For insulin tolerance tests, mice were fasted for 6 h before i.p. administration of 0.75 unit kg^−1^ of body weight of insulin. Blood glucose concentrations were measured before and after the injection at the indicated time points.

### Tissue metabolite extraction and gas chromatography/mass spectrometric analysis

Polar and non-polar metabolites were extracted from tissue using methanol/water/chloroform and derivitized as previously described^55^. Briefly, polar metabolites were derivatized to form methoxine-TBDMS derivatives by incubation with 2% methoxylamine hydrochloride dissolved in pyridine at 37 °C for 1 h followed by addition of N-tert-butyldimethylsilyl-N-methyltrifluoroacetamide (MTBSTFA) with 1% tert-butyldimethylchlorosilane (TBDMCS) incubated at 37 °C for 30–60 min. Nonpolar metabolites were saponified to FFAs and transesterified to form FA methyl esters by incubation with 2% H2SO4 in methanol at 50 °C for 1 h. Derivatized polar samples were analysed by gas chromatography–mass spectrometry using a DB-35MS column (30 m × 0.25 mm i.d. × 0.25 um) installed in an Agilent 7890B gas chromatograph (GC) interfaced with an Agilent 5977 A mass spectrometer. Lipid samples were analysed by gas chromatography–mass spectrometry using a Select FAME column (100 m × 0.25 mm i.d.) installed in an Aglient 7890A GC interfaced with an Agilent 5975C mass spectrometer.

### Tissue harvest and histology

Adipose tissue depots were carefully dissected to avoid contamination from surrounding tissue. Samples for RNA or protein were frozen down immediately in liquid nitrogen and then stored at −80 °C for further analysis. For histology, tissue pieces were fixed by 10% formalin. Embedding, sectioning and Hematoxylin & Eosin (HE) and PAS staining was done by the UMass Medical School Morphology Core. For Oil Red O staining, liver samples were embedded in OCT before sectioning and staining. For cell size measurements, 9–12 images were taken from each mouse (*n*=3 wild-type and 3 conditional KOs). Image J was used to measure cell size and the distribution of cell size as percentage of total counted cells was analysed.

### Western blots

Insulin stimulated signalling in each tissue was determined in mice that were fasting for 6 h before injection with 0.75 unit kg^−1^ of body weight of insulin for 15 min. Each tissue was collected and frozen down immediately in liquid nitrogen and then stored at −80 °C for subsequent lysis and western blots analysis with the indicated antibodies. Cells were lysed in a buffer containing 50 mM Hepes, pH 7.4, 40 mM NaCl, 2 mM EDTA, 1.5 mM NaVO4, 50 mM NaF, 10 mM sodium pyrophosphate, 10 mM sodium β-glycerophosphate and 1% Triton X-100 typically 16 h after the cells were replenished with fresh culture medium. Tissues were homogenized using a TissueLyser (Qiagen) in the same lysis buffer but additionally supplemented with 0.1% SDS, 1% sodium deoxycholate. An equal amount of total protein was loaded into acrylamide/bis-acrylamide gels and transferred to polyvinylidene fluoride membranes for detection with the indicated antibodies. Briefly, membranes were incubated with primary antibodies (1:1,000 dilution) in 5% milk/PBST or 5% BSA/PBST overnight. Horese radish peroxidase-conjugated secondary antibodies (1:3,000 dilution) were given for 1 h. Western blots were developed by enhanced chemiluminescence (PerkinElmer) and detected by X-ray films.

### Primary cell isolation and *in vitro* differentiation

SVF cells were isolated by digesting the sWAT of *Ubc;rictor* mice in digestion buffer (NaCl (123 mM), KCl (5 mM), CaCl2 (1.3 mM), glucose (5 mM), HEPES (100 mM), P/S (1%), BSA (4%), pH 7.4) containing collagenase A at 1.5 mg ml^−1^ (Roche). After 45 min of digestion, the digested tissue was filtered through 100 μm cell strainers (BD Falcon). Cells were collected by centrifugation (at 300*g*, 5 min) and cultured in DMEM (Invitrogen) supplemented with 10% FBS and penicillin/streptomycin at 37 °C. To induce *Rictor* deletion, *Ubc;rictor* SVF cells were treated with 1 μM 4-OHT or vehicle for 2 days when start to differentiate by adding differentiation medium containing 2 μg ml^−1^ dexamethasone, 100 nM insulin, 1 μM rosiglitazone and 0.5 mM IBMX for 2 days, and then induced with 100 nM insulin for another 2 days before changing back to regular medium. At different time points, the differentiated adipocytes were collected for protein, mRNA or Oil-Red-O staining analysis. For Oil Red O staining, the differentiated cells were washed three times with PBS and fixed with 10% buffered formalin at 4 °C overnight. Cells were then stained for 10 min at 37 °C with a filtered Oil Red O solution (0.5% Oil Red O in isopropyl alcohol), washed three times with distilled water, and visualized.

### Lipogenesis assay

Isolated SVF from Ubc;rictor mouse were cultured and differentiated into adipocytes for 7 days. The cells were then incubated in KRH buffer supplemented with 2.5% BSA and 2 μCi per ml D-[U-14C]-glucose (PerkinElmer) for 4.5 h with or without the presence of 150 nM insulin. The cells were lysed with Doley's solution (isopropyl alcohol:hexane:1N H2SO4 (v:v:v)=4:1:1), and newly synthesize lipids were extracted with hexane. About 1/3 of hexane phase was used for analysing triglycerides (TAGs), and the remaining were dried, deacylated with ethanol:water:KOH (v:v:v)=20:1:1 at 80 °C for an hour, neutralized with sulfuric acid and dissolved the neutral FFAs in hexane. The samples containing TAGs and FFAs were dried, reconstituted in scintillation fluid, and incorporation of 14C was determined by counting counts per minute (CPM). Each condition was done in duplicate, and the experiment was repeated with three independently isolated SVF. The incorporation was then normalized with protein content measured by BCA protein assay kit (Bio-Rad).

### 2-DOG glucose uptake

Isolated SVF from Ubc;rictor mouse were cultured and differentiated into adipocytes for 7 days. The cells were incubated in KRH buffer supplemented with 0.5% BSA and 2 mM sodium pyruvate, with or without 150 nM insulin stimulation for 15 min. [1, 2-^3^H] 2-deoxy-D-glucose (PerkinElmer) was added to the samples and incubate at 37 °C for 10 min, and the assays were terminated by three-time KRH wash. The cells were lysed with 1% Triton, dissolved in scintillation buffer and uptaken ^3^H was determined by counting CPM level. The 2-DOG uptake level was normalized with protein concentration of each sample. Each condition was done in triplicate.

### Primary hepatocytes isolation and culture

Hepatocytes were isolated from mice using a modified two-step perfusion method using Liver Perfusion Media and Liver Digest Buffer (Invitrogen). Cells were seeded on plates (pre-coated (1 h) with collagen I (BD Biosciences)) in DMEM plus 10% FBS, 2 mM sodiumpyruvate, 1 μM dexamethasone and 100 nM insulin plus 2% penicillin/streptomycin. After attachment (3 h), the medium was removed and the hepatocytes were incubated (22 h) in maintenance medium (DMEM (4.5 g l^−1^ glucose) supplemented with 10% FBS, 0.2% BSA, 2 mM sodium pyruvate, 2% Pen/Strep, 0.1 μM dexamethasone, 1 nM insulin) before stimulated with insulin at 100 nM insulin for 30 min.

### Measurement of lipolysis of differentiated adipocytes and adipose tissue

For measurement of lipolysis, differentiated adipocytes at day7 and pgWAT from mice were cultured in DMEM with or without isoproterenol at 10 μM for 4 or 6 h, respectively, before collecting medium to measure glycerol concentration using commercial kit (Sigma). The glycerol level was normalized with protein concentration of differentiated adipocytes and tissue mass of pgWAT.

### Immunohistochemistry and β-cell mass

Paraffin-embedded pancreatic sections were immunohistochemical stained with insulin. β-Cell mass of five mice per group of each genotype was measured in insulin-stained pancreas section using ImageJ (NIH, Bethesda, MD).

### Gene expression analysis

Cells or tissues were lysated with Qiazol (Invitrogen) and total RNA was isolated with the RNeasy kit (Invitrogen). Equal amounts of RNA were retro-transcribed to cDNA using a high-capacity cDNA reverse transcription kit (#4368813, Applied Biosystems). Quantitative real-time PCR was performed in 10 μl reactions using a StepOnePlus real-time PCR machine from Applied Biosystems using SYBR Green PCR master mix (#4309156, Applied Biosystems) according to manufacturer instructions. Relative mRNA expression was determined by the ΔCt method and Tbp expression was used as a normalization gene in all conventional PCR with reverse transcription experiments. Primer information is listed in (Supplementary Table 2).

### Statistics

Unless otherwise stated, values given are mean±s.e.m. Two-way analysis of variance was performed where indicated. For most experiments, unpaired two-tailed Student's *t*-tests was used to determine statistical significance among two groups (*P<0.05; **P<0.01;***P<0.001).

## Additional information

**How to cite this article**: Tang, Y. *et al*. Adipose tissue mTORC2 regulates ChREBP-driven *de novo* lipogenesis and hepatic glucose metabolism. *Nat. Commun.* 7:11365 doi: 10.1038/ncomms11365 (2016).

## Supplementary Material

Supplementary Information Supplementary Figures 1-10 and Supplementary Tables 1-2.
