# Supplementary material for: Adipose tissue mTORC2 regulates ChREBP-driven de novo lipogenesis and hepatic glucose metabolism
Source: Nat Commun. 2016 Apr 21;7:11365. doi: 10.1038/ncomms11365 (PMC4844681; doi:10.1038/ncomms11365)
Supplement: Supplementary Information — Supplementary Figures 1-10 and Supplementary Tables 1-2. [file ncomms11365-s1.pdf]

Supplementary Figure 1

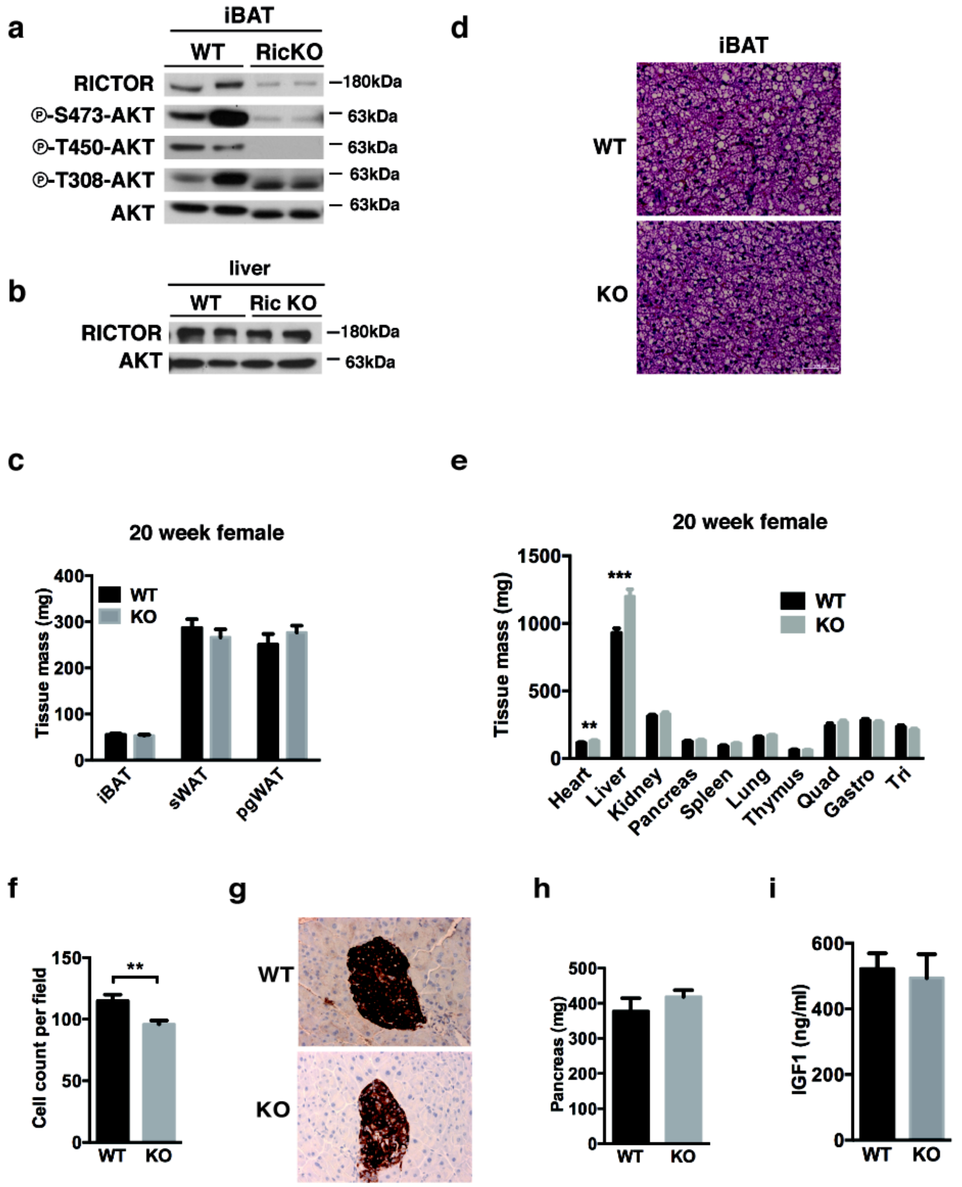

Supplementary Figure 1. The general phenotypes of adipose tissue *Rictor* knockout mice.

(a) Western blot of RICTOR, AKT and phospho-AKT in iBAT of $Rictor^{fl/fl}$ (WT) and $Rictor^{Adipoq\text{-}Cre}$ (KO) mice.

(b) Western blot of RICTOR and AKT in liver.

(c) Individual fat tissue mass of female mice at 20 weeks old. n=8-13.

(d) Representative images of H&E staining of iBAT.

(e) Individual lean tissue mass of female mice at 20 weeks old. n=8-13.

(f) Number of hepatocyte nuclei/field from liver H&E stained images. Four images from the liver of 4 mice were used in each group.

(g) Insulin staining of pancreas.

(h) Pancreas mass from samples used for in (g) and different from Figure 1(g). n=5.

(i) Plasma IGF1 level. n=6. Data were analyzed by Student's t test. Values are expressed as mean+sem. $*p < 0.05$; $**p < 0.01$; $***p < 0.001$. Scale bar: 100μM.

Supplementary Figure 2

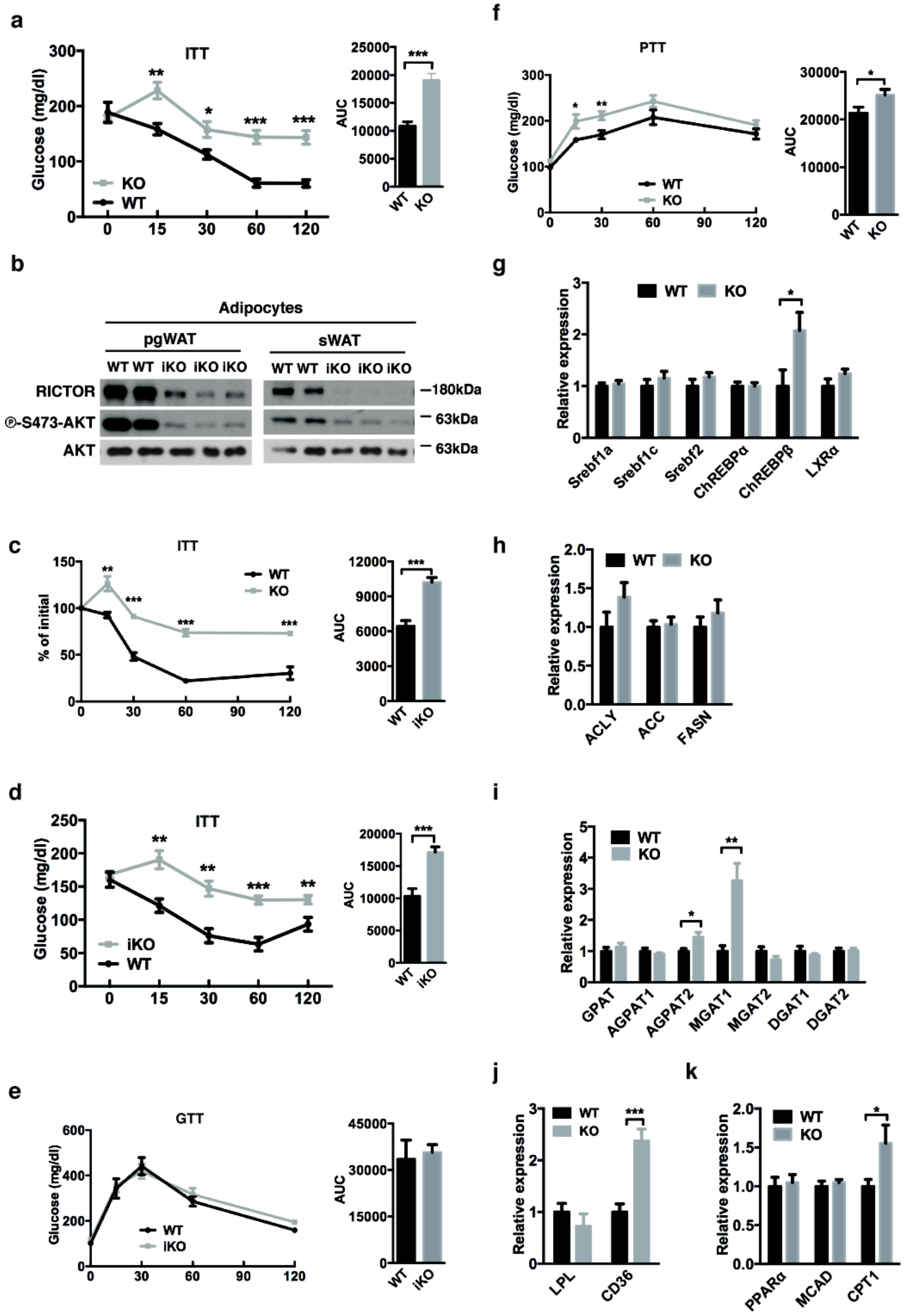

Supplementary Figure 2. Insulin resistance of inducible adipose tissue *Rictor* knockout mice.

(a) ITT of female *Rictor*$^{fl/fl}$ (WT) and *Rictor*$^{Adipoq\text{-}Cre}$ (KO) mice 8 weeks. n=6 or 8.

(b) Western blot of RICTOR, AKT and phosphorylation of AKT at S473 of isolated adipocytes from *Rictor*$^{fl/fl}$ (WT) and inducible *Rictor*$^{Adipoq\text{-}CreERT2}$ (iKO) mice at 3 weeks post first tamoxifen injection.

(c) ITT of mice at 5 weeks post first tamoxifen injection. n=6 or 8.

(d) The raw data of (c).

(e) GTT of mice at 6 weeks post first tamoxifen injection. n=6 or 8.

(f) Pyruvate tolerance test (PTT) of *Rictor*$^{fl/fl}$ (WT) and *Rictor*$^{Adipoq\text{-}Cre}$ (KO) male mice at 10 weeks old. n=7 or 8.

(g-k) The relative mRNA expression of indicated genes in liver of *Rictor*$^{fl/fl}$ (WT) and *Rictor*$^{Adipoq\text{-}Cre}$ (KO) mice. n=8. Data were analyzed by Student's t test. Values are expressed as mean+sem. $^{*}p < 0.05$; $^{**}p < 0.01$; $^{***}p < 0.001$.

Supplementary Figure 3

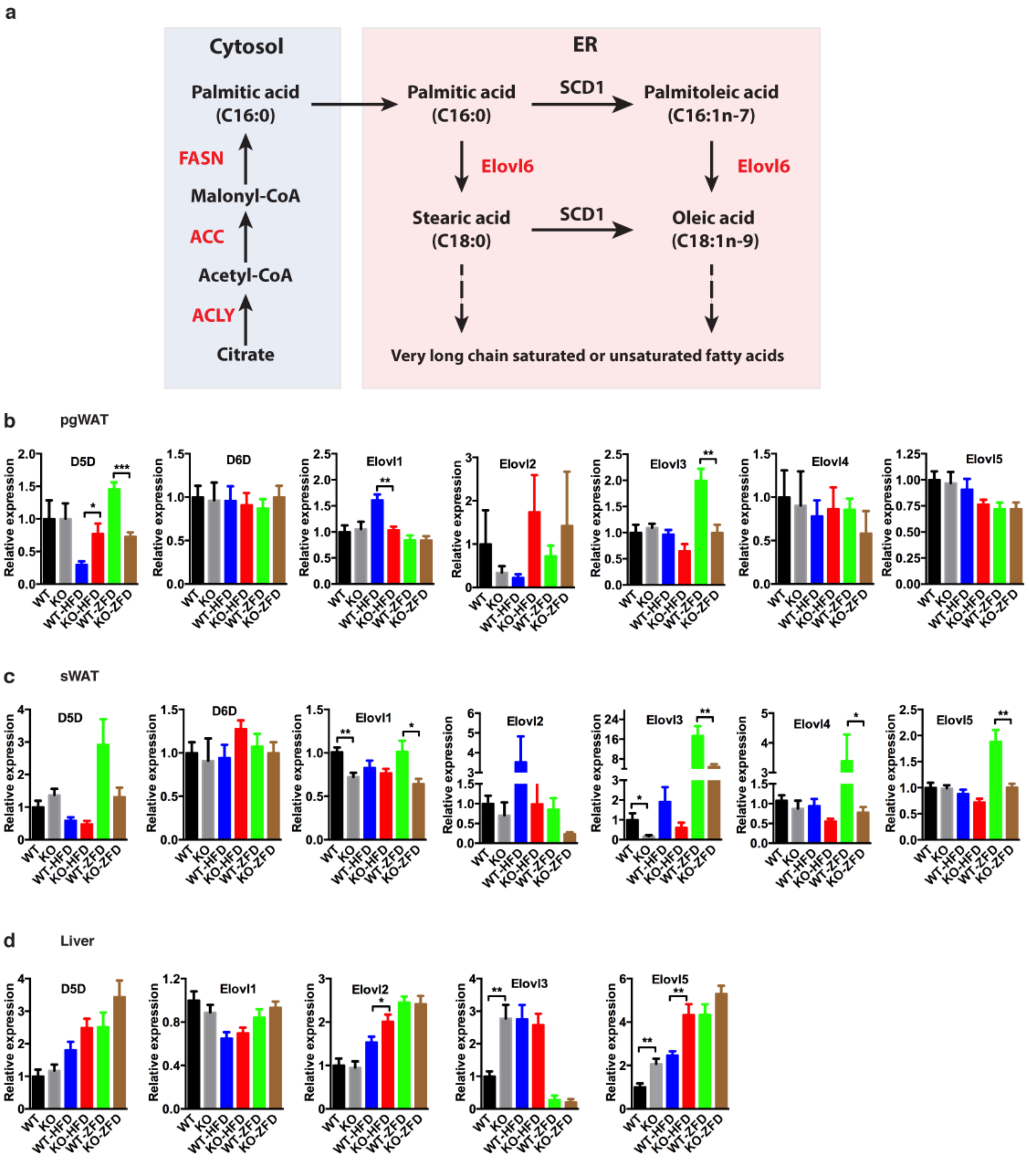

Supplementary Figure 3. The role of adipose mTORC2 in DNL and elongation.
(a) Model of DNL and elongation in adipose tissue. Genes with red color are regulated by adipose tissue mTORC2.
(b-d) The relative mRNA expression of indicated genes in pgWAT (b), sWAT (c) and liver (d) of rictor$^{fl/fl}$ (WT) and *Rictor*$^{Adipoq\text{-}Cre}$ (KO) under chow, HFD or ZFD. n=8. Data were analyzed by Student’s t test. Values are expressed as mean+sem. *$p < 0.05$; **$p < 0.01$; ***$p < 0.001$.

Supplementary Figure 4

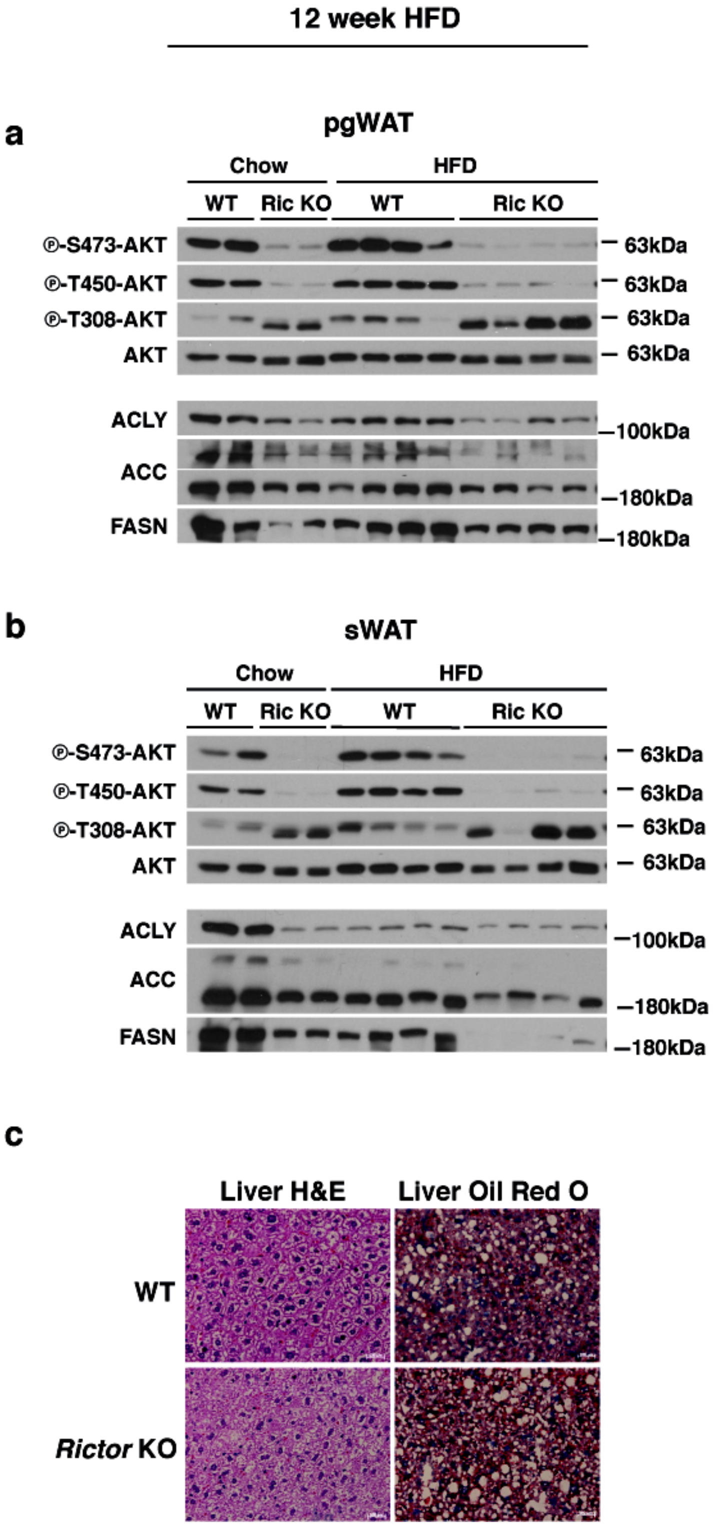

Supplementary Figure 4. HFD represses DNL in WAT and induces fatty liver.
(a-b) Western blot of indicated proteins of pgWAT (a) and sWAT (b) of *Rictor*$^{fl/fl}$ (WT) and *Rictor*$^{Adipoq\text{-}Cre}$ (KO) mice under chow and HFD.
(c) The representative images of liver H&E staining and liver Oil Red O staining of *Rictor*$^{fl/fl}$ (WT) and *Rictor*$^{Adipoq\text{-}Cre}$ (KO) mice under HFD for 12 weeks. Scale bar: 100μM.

Supplementary Figure 5

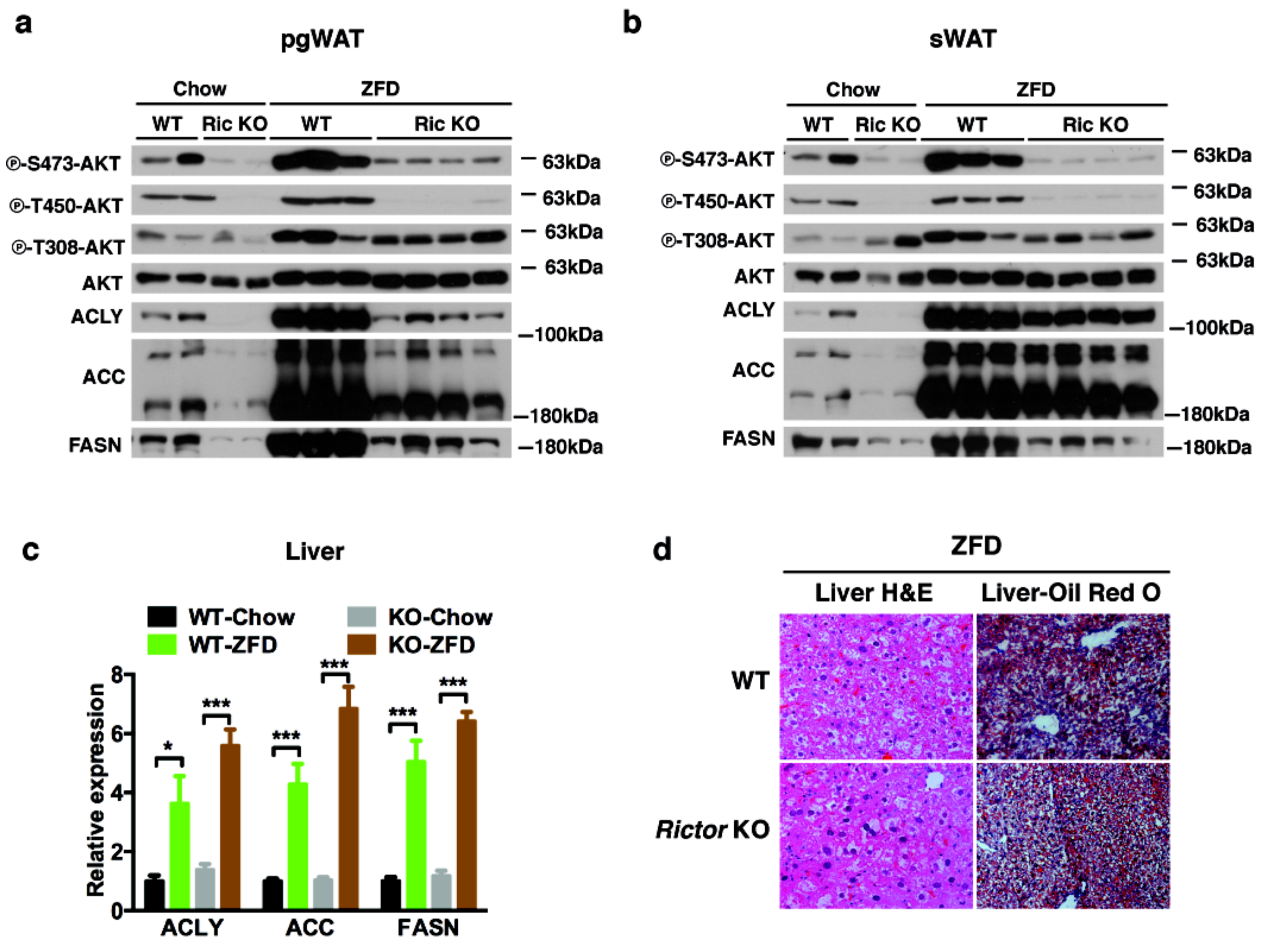

Supplementary Figure 5. ZFD increases hepatic DNL and causes fatty liver.
(a-b) Western blot of indicated proteins in pgWAT (a) and sWAT (b) of *Rictor*$^{fl/fl}$ (WT) and *Rictor*$^{Adipoq\text{-}Cre}$ (KO) mice under chow and ZFD for 12 weeks started at 8 weeks old.
(c) The relative mRNA expression of *acly*, *acc* and *fasn* in liver of mice under ZFD for 12 weeks. n=8. Data were analyzed by Student's t test between groups. Values are expressed as mean+sem.
(d) The representative images of liver H&E staining and liver Oil Red O staining of mice under ZFD for 12 weeks. *p < 0.05; **p < 0.01; ***p < 0.001. Scale bar: 100μM.

Supplementary Figure 6

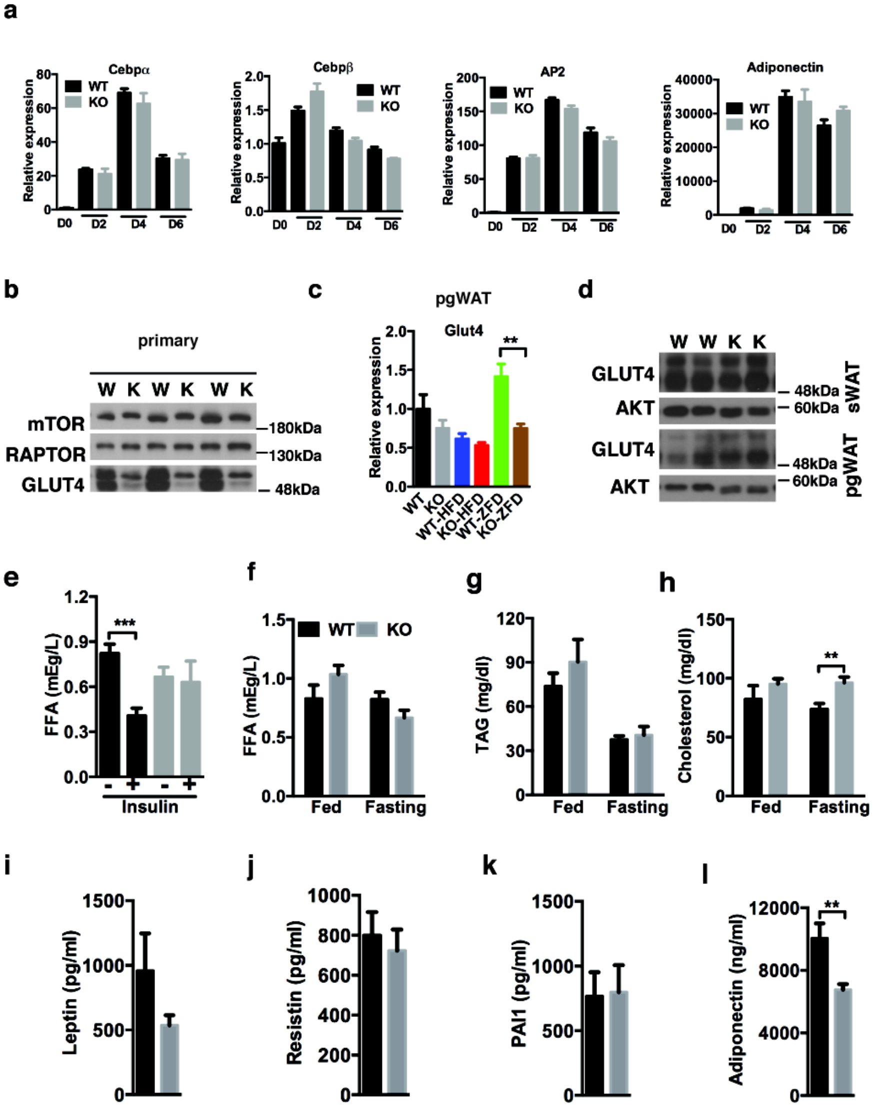

Supplementary Figure 6. Plasma metabolites of adipose tissue *Rictor* knockout mice

(a) The relative mRNA expression of indicated genes in differentiated cells at various time points. n=3.

(b) The expression of indicated proteins in differentiated adipocytes. n=3.

(c) The relative expression of *Glut4* gene in pgWAT of *Rictor*$^{fl/fl}$ (WT) and *Rictor*$^{Adipoq\text{-}Cre}$ (KO) mice. n=8.

(d) The GLUT4 protein level measured by western blotting in sWAT and pgWAT.

(e) The plasma FFA level after 6 hour fasting with or without insulin injection for 15 minutes.

(f) The plasma FFA level under random feeding and 6 hour fasting.

(g) The plasma TAG level under random feeding and 6 hour fasting.

(h) The plasma cholestorol level under random feeding and 6 hour fasting.

(i) The plasma leptin level under random feeding.

(j) The plasma resistin level under random feeding.

(k) The plasma PAI1 level under random feeding.

(l) The plasma adiponectin level under random feeding. n=6. Data were analyzed by Student's t test. Values are expressed as mean+sem. $^*p < 0.05$; $^{**}p < 0.01$; $^{***}p < 0.001$.

Supplementary Figure 7 Original Films

a

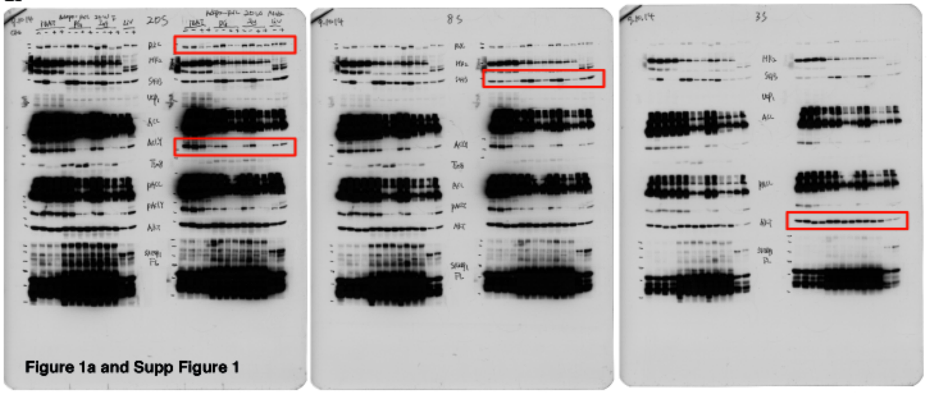

b

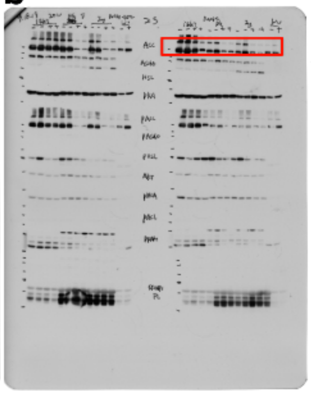

c

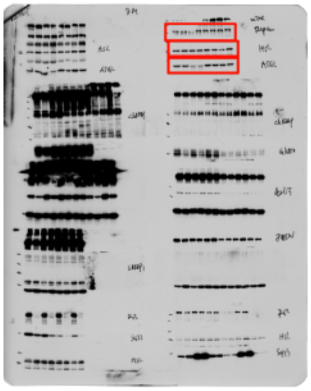

d

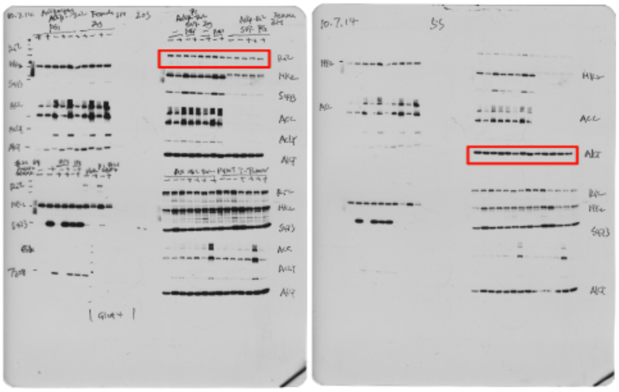

Supplementary Figure 8 Original Films

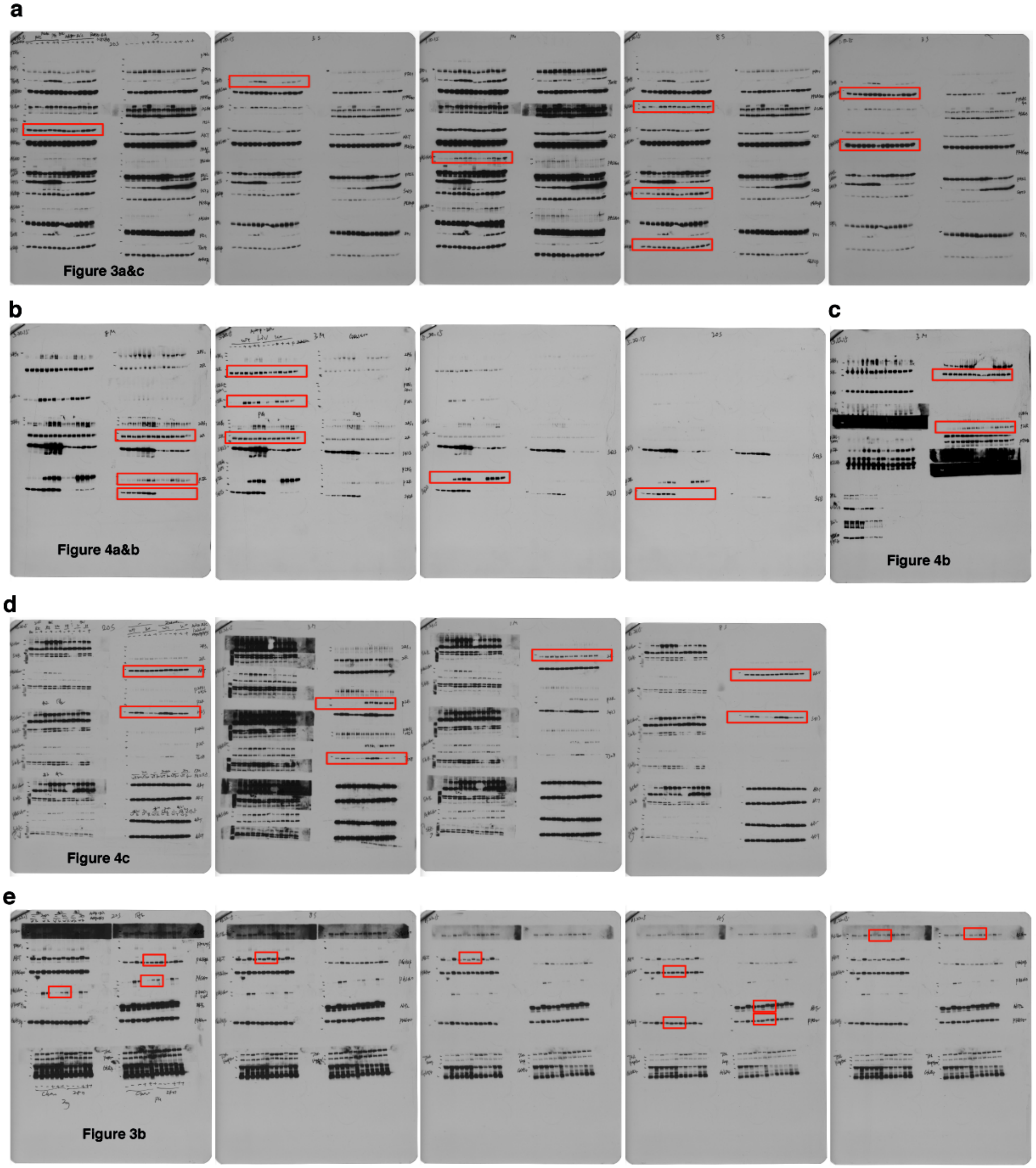

Supplementary Figure 9 Original Films

a

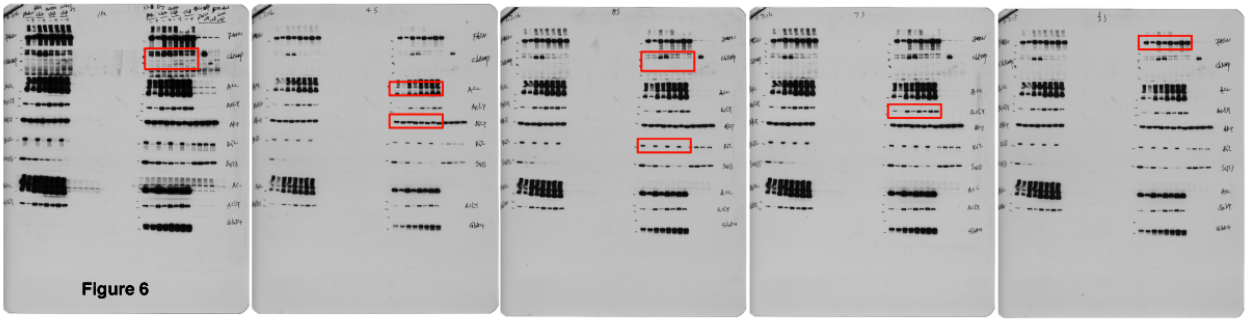

b

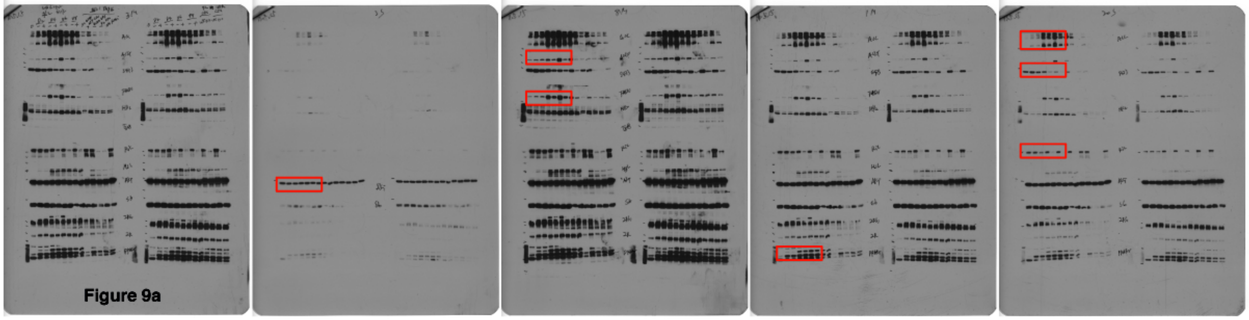

c

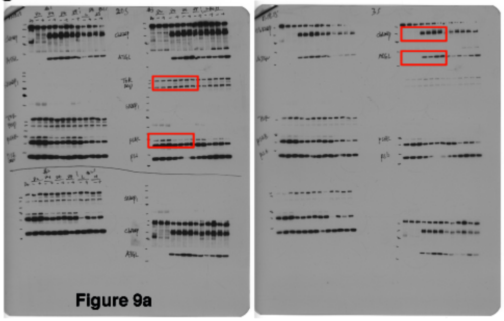

d

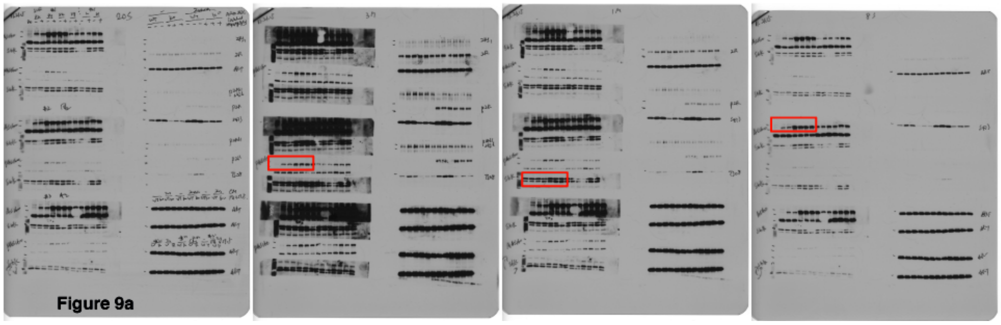

Supplementary Figure 10 Original Films

a

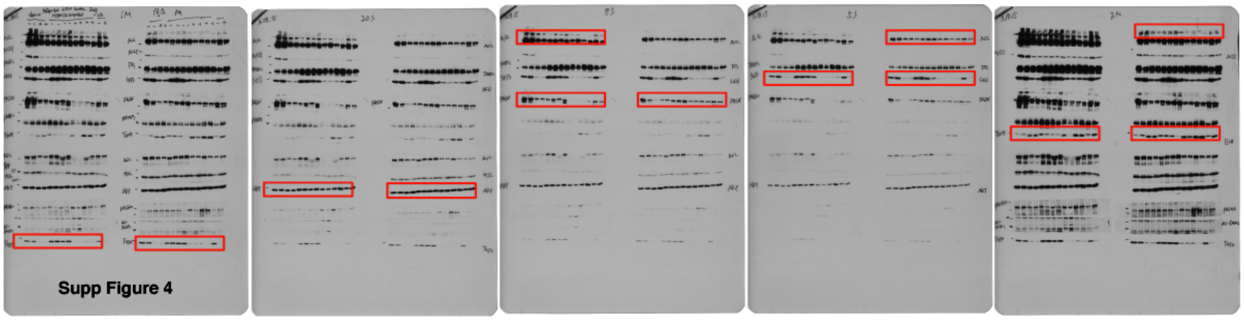

b

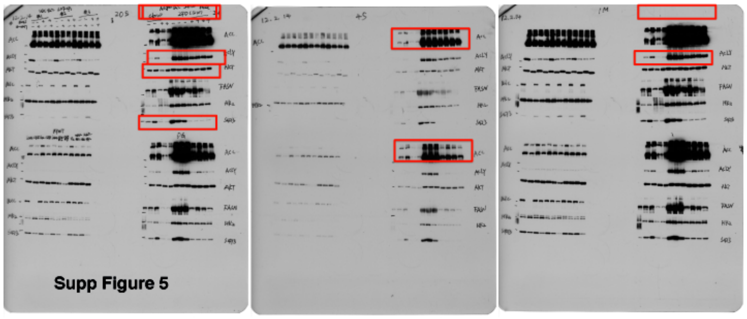

c

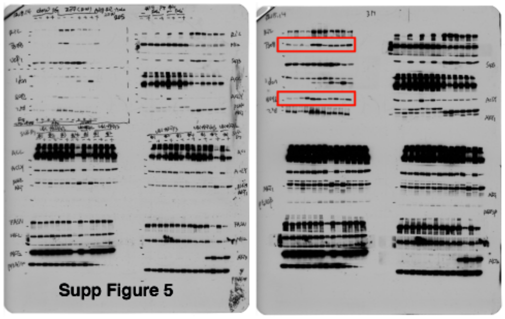

d

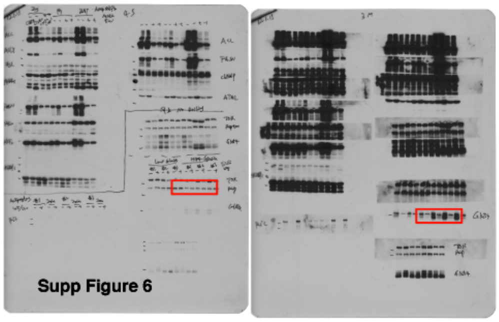

Supplementary Table 1

A comparison between the *Rictor*$^{Adipoq\text{-}Cre}$ mice described in this study and previously described *Rictor*$^{aP2\text{-}Cre}$ mice

**Parameter Examined**	**Observed difference relative to control**	
	***Rictor*$^{Adipoq\text{-}Cre}$**	***Rictor*$^{aP2\text{-}Cre}$***
Total body mass	**No difference**	**Increased**
Adipose tissue mass	No difference	No difference
Liver mass	**Increased**	**No difference**
Hepatic TAGs	Slightly Increased	Normal/Slightly increased
Lean tissue mass	**No difference**	**Increased**
Circulating IGF-1	**No difference**	**Increased/normal**
Circulating Insulin	Elevated	Elevated
Leptin	Normal	Normal
Adiponectin	Decreased	Normal/Decreased
Islet mass	**No difference**	**Increased**
ITT	**Severe Insulin Resistance**	**Normal/Slight Insulin resistance**
GTT	Normal	Normal/Resistance (aged mice)
Muscle glucose uptake (clamp)	**Normal**	**Decreased**
Fat glucose uptake (clamp)	Decreased	Decreased
HGP (clamp)	Increased	Increased
GIR (clamp)	Decreased	Decreased
AS160 phosphorylation	**Normal**	**Decreased**
FOXO1 phosphorylation	**Normal**	**Decreased**
Basal fat lipolysis (ex vivo)	Elevated	Elevated
Basal fat lipolysis (primary cell)	Normal	n.d.
Response to HFD	**Reduced adipose tissue growth**	**Increased adipose tissue growth**
Response to ZFD	Reduced adipose tissue growth	n.d.
Chrebpβ expression in WAT	Decreased	n.d.
DNL genes in WAT	Decreased	n.d.
Elovl6 expression in WAT	Decreased	n.d.
Scd1 expression in WAT	normal	n.d.
Lipid composition (fat)	Altered	n.d.
Lipid composition (liver)	Altered	n.d.

* *aP2-Cre;Rictor* mice are described in Reference 17 & 18 of the Main text

BOLD Indicates key differences between models
n.d. Not determined
ITT Insulin tolerance test
GTT Glucose tolerance test
HGP Hepatic glucose production
GIR Glucose infusion rate
HFD High fat diet
ZFD Zero fat diet
DNL De novo lipogenesis
WAT White adipose tissue

Supplementary Table 2. Primer sequences for quantitative RT-PCR analysis

Gene	Forward primer (5'-3')	Reverse primer (5'-3')
Tbp	GAAGCTGCGGTACAATTCCAG	CCCCTTGTACCCTTCACCAAT
Pparγ	TCAGCTCTGTGGACCTCTCC	ACCCTTGCATCCTTCACAAG
Srebf1a	TAGTCCGAAGCCGGGTGGGCGCCGG	GATGTCGTTCAAAACCGCTGTGTGT C
Srebf1c	AAGCAAATCACTGAAGGACCTGG	AAAGACAAGCTACTCTGGGAG
Srebf2	GGATCCTCCCAAAGAAGGAG	TTCCTCAGAACGCCAGACTT
Chrebpα	CGACACTCACCCACCTCTTC	TTGTTCAGCCGGATCTTGTC
Chrebpβ	TCTGCAGATCGCGTGGAG	CTTGTCCCGGCATAGCAAC
Acly	CTCACACGGAAGCTCCATAA	ACGCCCTCATAGACACCATC
Acc	GGAGATGTACGCTGACCGAGAA	ACCCGACGCATGGTTTTCA
Fasn	GCTGCGGAAACTTCAGGAAAT	AGAGACGTGTCACTCCTGGACTT
Elvol6	TCAGCAAAGCACCCGAAC	AGCGACCATGTCTTTGTAGGAG
Scd1	CCCTGCGGATCTTCCTTATC	TGTGTTTCTGAGAACTTGTGGTG
Elovl1	GTGGCCCAGCCCTACCTT	TGTGCAGTGAGACCAGGACAA
Elovl2	TCAATGCTTTCTTGGACAACATG	GGTAAGAGTCCAGCAGGAACCA
Elovl4	GTGGGTGGCTGGAGGCCAAGCGTTTT TCGG	ATTCGTGGCCGTCTTTCCGGTTTTT GACTG
D6D	TATGGCAAGAAGAAGCTGAAATACCTG CCC	TGCTTACCTCCATAAATGAAGCTGC CGTCC
D5D	GGGTGGACTTGGCCTGGATGCTCAGC TTCT	GCTGCTATTGGTGAAGGTAAGCGT CCAACC
Elovl5	CCCCCGAGATACAAGAGTCA	GCAGGGTGAGTCCAAGGTTA
Elovl3	TCCGCGTTCTCATGTAGGTCT	GGACCTGATGCAACCCTATGA
